# Proteomic analysis of the *Treponema pallidum* subsp. *pallidum* SS14 strain: coverage and comparison with the Nichols strain proteome

**DOI:** 10.3389/fmicb.2024.1505893

**Published:** 2024-12-11

**Authors:** Simon Houston, Steven Marshall, Alloysius Gomez, Caroline E. Cameron

**Affiliations:** ^1^Department of Biochemistry and Microbiology, University of Victoria, Victoria, BC, Canada; ^2^Department of Medicine, Division of Allergy and Infectious Diseases, University of Washington, Seattle, WA, United States

**Keywords:** *Treponema pallidum*, syphilis, proteomics, outer membrane proteins, vaccine candidates

## Abstract

**Introduction:**

Strains of the syphilis spirochete, *Treponema pallidum* ssp. *pallidum*, group into one of two deep-branching clades: the Nichols clade or the globally dominant Street Strain 14 (SS14) clade. To date, in-depth proteome-wide analyses have focused on Nichols clade strains.

**Methods:**

The *T. pallidum* SS14 clade reference strain (SS14) proteome was characterized via protein detection and quantification analyses using mass spectrometry, and comparison was made to the Nichols clade reference strain (Nichols) proteome.

**Results:**

Approximately two thirds of all proteins from *T. pallidum* SS14 were detected and quantitated, allowing confirmation of expression of 259 proteins for the first time in this strain, including 11 known/putative outer membrane proteins (OMPs). SS14 and Nichols proteome comparative analyses demonstrated similar protein expression/quantification profiles between the two strains, and showed that inter-strain amino acid sequence differences are located primarily within predicted surface-exposed regions in 16 known/putative OMPs.

**Discussion:**

This study provides the first comparative analyses of the proteomes from the *T. pallidum* SS14 and Nichols strains. The findings inform syphilis vaccine design by confirming the expression of known/predicted OMP vaccine candidates in SS14 treponemes, and via the finding that most inter-strain variable residues found in OMPs are predicted to be located in surface-exposed, host-facing regions of these proteins.

## Introduction

*Treponema pallidum* ssp. *pallidum* (hereafter *T. pallidum*), the causative agent of infectious and congenital syphilis, continues to be a global public health concern, with rates increasing around the world (Herbert and Middleton, 2012; Savage et al., 2012; Chen et al., 2017; Arrieta and Singh, 2019; Korenromp et al., 2019). Furthermore, syphilis infections increase the risk of transmission and acquisition of other STIs, including HIV (Nusbaum et al., 2004; Douglas, 2009).

Comparative genomics studies have demonstrated that the worldwide distribution of *T. pallidum* strains consists of two deep-branching phylogenetic clades, with each clade named after their respective reference strain: Nichols and SS14 clades (Mikalova et al., 2010; Smajs et al., 2012; Petrosova et al., 2013; Nechvatal et al., 2014; Arora et al., 2016). The Nichols strain, originally isolated in Washington D.C. in 1912 from the cerebrospinal fluid of a patient with secondary syphilis (Nichols and Hough, 1913), is a laboratory reference strain that has undergone continuous passage in the rabbit animal model (LaFond and Lukehart, 2006), and more recently the *in vitro* culture system (Edmondson et al., 2018; Edmondson et al., 2021; Edmondson and Norris, 2021), since its isolation. The SS14 strain was first isolated in 1977 in Atlanta, Georgia from a skin lesion of an individual with secondary syphilis, and thus represents a more recently isolated clinical strain that has undergone fewer passages through the animal model or *in vitro* culture system (Stamm et al., 1983; Stamm et al., 1988). Based on currently available clinical sampling data, strains belonging to the SS14-like clade dominate syphilis infections worldwide (Arora et al., 2016; Beale et al., 2021; Lieberman et al., 2021).

Molecular typing and genomics studies have identified genetic differences between strains from the SS14 and Nichols clades (Petrosova et al., 2013; Nechvatal et al., 2014; Lieberman et al., 2021). Many of these genetic differences are conserved between strains within each clade, and are predicted to result in amino acid sequence differences in the corresponding encoded proteins (Mikalova et al., 2010; Smajs et al., 2012; Petrosova et al., 2013; Nechvatal et al., 2014; Arora et al., 2016; Lieberman et al., 2021). Included in these proteins are known and potential outer membrane proteins (OMPs) (Mikalova et al., 2010; Smajs et al., 2012; Petrosova et al., 2013; Nechvatal et al., 2014; Arora et al., 2016; Lieberman et al., 2021), a protein class that is the focus of current efforts for syphilis vaccine development.

In the present study, the proteome of *in vitro*-cultured *T. pallidum* SS14 was characterized using mass spectrometry (MS), providing the detection and quantification of proteins encoded by *T. pallidum* SS14 (referred to as proteome-wide analyses of SS14). Comparative proteomic analyses of SS14 and Nichols strains revealed shared protein expression and quantification profiles across all detected proteins and also among protein classes of specific interest, and validated inter-strain protein sequence variances identified by genome sequencing. Most of the SS14-Nichols inter-strain protein sequence variances found in the treponemal OMPs were mapped to predicted surface-exposed *T. pallidum* protein regions. This finding has implications for syphilis vaccine design as the treponemal OMPs are located at the pathogen-host interface and are targeted by the host immune response.

## Materials and methods

### *In vitro* culture of *Treponema pallidum*

*Treponema pallidum* (SS14 strain; supplied as a frozen stock by Lorenzo Giacani, University of Washington, Seattle, WA (Romeis et al., 2021)) was used for *in vitro* culture and sub-culture of treponemes in the presence of Sf1Ep (NBL-11) cottontail rabbit epithelial cells (ATCC CCL-68; American Type Culture Collection [ATCC], Rockville, MD, USA), as previously described (Edmondson et al., 2018; Edmondson et al., 2021; Edmondson and Norris, 2021; Houston et al., 2024). This SS14 reference strain was originally isolated by John Clark (Sexually Transmitted Disease Laboratory Program of the Centers for Disease Control) in 1977 in Atlanta, Georgia from an individual with secondary syphilis (Stamm et al., 1983). Trypsin-free dissociation buffer (Edmondson and Norris, 2021) was used to maintain OMP integrity during *T. pallidum* dissociation steps. The final *in vitro*-cultured *T. pallidum* sample contained 2.3 × 10^9^ treponemes suspended in sterile 0.9% saline (0.9% w/v NaCl, pH 7.0).

### *Treponema pallidum* SS14 protein sample preparation: present study

In the present study, the *T. pallidum* SS14 protein sample was prepared for MS using a previously optimized sample preparation workflow comprised of three main steps: (1) centrifugation steps to remove contaminants, (2) lysis steps to extract *T. pallidum* proteins, and (3) a final centrifugation step to isolate soluble *T. pallidum* proteins. In step (1), insoluble rabbit gross cellular debris was removed from *in vitro*-cultured *T. pallidum* via slow-speed (220 × *g*) centrifugation steps. High-speed (17,000 × *g*) centrifugation steps were then performed to remove soluble contaminants. In step (2), chemical lysis of the *T. pallidum* pellets was performed via suspension in lysis buffer (50 mM ammonium bicarbonate pH 8.0, 0.9% sodium deoxycholate [Sigma-Aldrich Canada Co., Oakville, ON, Canada]) for 30 min on ice, followed by physical lysis using ultrasonication. In step (3), high speed centrifugation (17,000 × *g*) was used to remove precipitated, aggregated, or insoluble proteins and cellular debris. Further details are provided in our prior *T. pallidum* proteomics studies (Houston et al., 2023; Houston et al., 2024).

### *Treponema pallidum* protein sample preparations from previous studies used for data integration

The combined *T. pallidum* SS14 proteome coverage was determined by integrating MS data from the present study results with the MS data from a previous *T. pallidum* SS14 genetic engineering study (Romeis et al., 2021). In the previous study by Romeis et al., the authors cultured wild-type and genetically engineered *T. pallidum* SS14 under *in vitro* culture conditions and prepared protein samples by pelleting 1 × 10^9^ treponemes using high speed centrifugation, followed by SDS-PAGE analysis, in-gel trypsin digestion, and liquid chromatography-tandem MS (LC-MS/MS) analysis using an LTQ HP1100 mass spectrometer. Additional details on the previous study can be found in Romeis et al. (2021). For the proteome comparative analyses of *T. pallidum* SS14 and Nichols Strains, we compared the SS14 strain MS data from the present study with MS data derived from our previous *T. pallidum* Nichols proteome study (Houston et al., 2024). In this previous study, three *in vitro*-cultured *T. pallidum* Nichols samples (containing 1.0 × 10^9^, 1.0 × 10^9^, and 1.55 × 10^9^ treponemes) were prepared using the optimized proteomics protocol, analyzed by LC–MS/MS, and analyzed for protein detection and label free quantification (LFQ) analyses as outlined in the current study.

### MS sample preparation and LC-MS/MS

MS sample preparation and LC–MS/MS were performed on the *T. pallidum* protein sample using the method outlined in previous work (Houston et al., 2024) which was comprised of 3 major steps: (1) in-solution trypsin digestion, (2) high-pH reversed phase fractionation, and (3) protein detection via LC–MS/MS. In step (1), in-solution trypsin digestion was performed on the *T. pallidum* protein sample as previously described (Houston et al., 2024), with the following modifications. Dithiothritol (Sigma-Aldrich Canada Co., Oakville, ON, Canada) was added to a final concentration of 20 mM to reduce disulphide bonds. Cleanup of the trypsin-digested protein sample was performed via centrifugation for 5 min at 17,000 x g followed by lyophilization of the sample supernatant. To reduce sample complexity (step 2), high-pH reversed phase fractionation was used to separate the trypsin-digested *T. pallidum* sample into 24 fractions based on hydrophobicity, as previously described (Houston et al., 2024), with the following changes. Buffer A was comprised of 10 mM ammonium hydroxide, pH 9.0. Buffer B consisted of 80% acetonitrile and 10 mM ammonium hydroxide, pH 9.0. The 24 concatenated fractions were speed vacuum concentrated, and diluted in 2% acetonitrile/ 0.1% formic acid (100 μL final volume per fraction). In step (3), fractionated samples were subjected to LC–MS/MS for protein detections, as previously described (Houston et al., 2024). Briefly, a 10 μL aliquot of each concatenated fraction was separated by on-line reversed phase liquid chromatography using a Thermo Scientific EASY-nLC 1,000 system with an Acclaim PepMap100 reversed-phase pre-column C18 (100 μm I.D., 2 cm length, 5 μm bead size, 100 Å pore size) (Thermo Fisher Scientific, San Jose, CA), and an AcclaimPepMap100 C-18 reversed phase nano-analytical column (75 μm I.D., 15 cm length, 3 μm bead size, 100 Å pore size) (Thermo Fisher Scientific, San Jose, CA), at a flow rate of 300 nL/min. The chromatography system was coupled on-line with an Orbitrap Fusion Tribrid mass spectrometer (Thermo Fisher Scientific, San Jose, CA, USA) equipped with a Nanospray Flex NG source (Thermo Fisher Scientific). Tryptic peptides were separated using a 120-min gradient of solvent A (A; 2% acetonitrile/0.1% formic acid) and solvent B (B; 90% acetonitrile/0.1% formic acid), comprised of the following steps: (i) 0–100 min, gradient change from 95% A / 5% B to 58% A / 42% B, (ii) 100–115 min, gradient change from 58% A / 42% B to 0% A / 100% B, and (iii) 115–120 min, gradient held at 0% A / 100% B. The Orbitrap Fusion Tribrid mass spectrometer instrument parameters (Fusion Tune 3.5 software) were set as outlined before (Houston et al., 2024).

### Protein detection: parameters and data analysis

Data analyses and the parameters used for protein detections were performed as described in our previous studies (Houston et al., 2023; Houston et al., 2024). In brief, tandem mass spectra were extracted using Proteome Discoverer version 3.0. (Thermo Scientific). Charge state deconvolution was not performed. All MS/MS samples were analyzed using Sequest (Thermo Fisher Scientific, San Jose, CA, USA; (version IseNode in Proteome Discoverer 3.0.0.757) containing either (1) a *T. pallidum* database (*T. pallidum* proteins, Nichols strain, NCBI reference sequence NC_021490, all proteome annotation revisions from June 17th 2013 – July 4th 2021), or (2) a *T. pallidum* SS14 database (*T. pallidum* proteins, SS14 strain, NCBI reference sequence: NC_021508, all proteome annotations from March 12th 2023). Proteome annotations from March 2023 were used in the SS14 database as these were the most up-to-date annotations available on the NCBI website when the present study was performed. Both databases also contained all reviewed (Swiss-Prot) canonical rabbit proteins (UniProt *Oryctolagus cuniculus* proteome, UP000001811) (Supplementary Table S1). Peptide/protein searches also included common contaminants.^1^ Protein detections in Scaffold (version Scaffold_5.1.2; Proteome Software Inc., Portland, OR, USA) were accepted if they could be established at 99.9% probability. Protein probabilities were assigned by the Protein Prophet algorithm (Nesvizhskii et al., 2003). Proteome coverage was calculated based on *T. pallidum* SS14 strain (NCBI reference sequence NC_021508, March 2023 annotation, 987 proteins); 981 proteins from predicted protein-coding genes, and six proteins potentially encoded by “pseudo genes.”

### Label free quantification

Label free quantification (LFQ) was performed in Scaffold (version Scaffold_5.2.2) using total spectral counts as a relative quantitative measure of protein abundances in SS14 and Nichols strains. Only protein detections that were derived from the *T. pallidum* SS14 database search in the present study were used for LFQ analyses. Sample 2 protein detections from the *in vitro*-cultured *T. pallidum* Nichols strain (Houston et al., 2024) were used for SS14-Nichols comparative analyses, as this sample contained the highest number of detected treponemal proteins. The default settings for the quantitative analysis setup in Scaffold were used. Normalized total spectral counts were selected for each protein, and proteins were ranked from most abundant (highest spectral count) to least abundant (lowest spectral count). The *T. pallidum* SS14 and Nichols databases, as described above, were used for searches, peptide detections, and protein quantifications. Scaffold search parameters were as follows: precursor mass tolerance = 10 ppm; fragment mass (MS/MS) tolerance = 0.6 Da; enzyme specificity = trypsin, with a maximum of two missed cleavages allowed; fixed modification = carbamidomethylation (C); variable modifications = acetylation of the peptide N-terminus and oxidation (M).

### Identification of SS14 proteome annotation errors (proteins prematurely truncated at the N-termini)

The *T. pallidum* database containing all proteome annotation revisions from June 17th 2013 to July 4th 2021 was used to identify SS14 proteins that have been annotated in the NCBI proteome with prematurely truncated N-termini, as described previously (Houston et al., 2023; Houston et al., 2024). This was achieved via the detection and identification of SS14 peptides that are not present in the SS14 proteome, but which are annotated as being located within extended N-terminal regions of proteins in previous versions of orthologous proteins from *T. pallidum* Nichols strain. Previously annotated versions of SS14 orthologs with similar extended N-termini (compared to the 2023 annotations) that also contained the detected SS14 peptides were then identified by searching previous revisions of the NCBI SS14 proteome annotations.

### Proteome-wide functional categorization of *Treponema pallidum* proteins

All treponemal protein sequences were assigned to functional categories based on the “Clusters of Orthologous Genes” (COG) (Tatusov et al., 1997) categories,^2^ as previously described (Houston et al., 2024).

### Data integration: combined *Treponema pallidum* SS14 proteome coverage

In a previous *T. pallidum* genetic engineering study, MS was used to confirm the expression of a 31 kDa protein produced from a genetically modified target gene (Romeis et al., 2021) in *T. pallidum* SS14, via the detection of proteins in the ~20–45 kDa size range. These MS analyses were performed as a genetic engineering confirmatory method (Romeis et al., 2021). For the MS analyses, the authors of the genetic engineering study analyzed a single sample prepared from the wild-type control SS14 strain and a single sample from a genetically engineered SS14 strain (Romeis et al., 2021). A beneficial consequence of these MS findings for the *T. pallidum* proteomics research field is the potential integration of these data with other *T. pallidum* MS studies focused on improving the proteome coverage of the SS14 strain. Therefore, to determine the combined *T. pallidum* SS14 proteome coverage in the present study, we integrated the MS data from the previous *T. pallidum* SS14 genetic engineering study (Romeis et al., 2021) with the *T. pallidum* SS14 MS results from the current study. For these MS data integration analyses, all detected *T. pallidum* proteins from the genetically engineered and wild-type control SS14 strains (Romeis et al., 2021) were used for determining the combined *T. pallidum* SS14 proteome coverage in the present study.

### Data integration: proteome-wide comparative analyses of SS14-Nichols inter-strain protein sequence differences

Proteome-wide comparative analyses of SS14 (NC_021508) and Nichols (NC_021490) protein annotations were conducted to identify treponemal proteins with at least one amino acid difference between the strains. Mass spectrometry-based proteomics analyses were then used to experimentally validate the expression of as many of these differences as possible by checking for detection of peptides containing these variations in our present SS14 study, the previous study that performed MS analysis to confirm genetic engineering of *T. pallidum* (Romeis et al., 2021), and additional previously performed *T. pallidum* proteomics studies (Osbak et al., 2016; Houston et al., 2023; Houston et al., 2024). Manual inspection of these peptides was used to confirm inter-species protein differences. AlphaFold 3^3^ (Abramson et al., 2024) was used for structure modeling of predicted/known OMPs with annotated and experimentally validated inter-strain amino acid differences, as detailed in below.

### Structure modeling of predicted/known OMPs with annotated inter-strain amino acid differences

Structure modeling was performed using AlphaFold 3 (see footnote 3) (Abramson et al., 2024). The mature amino acid sequences of known/predicted OMPs from SS14 (NCBI reference sequence NC_021508, March 2023 annotation) and the corresponding proteins from Nichols (NCBI reference sequence NC_021490, March 2023 annotation) were generated using SignalP 6.0^4^ (Teufel et al., 2022) and then submitted to the AlphaFold 3 (see footnote 3) server. The coordinate files for the highest confidence structure models were downloaded for visualization, labelling, and comparative analyses in the molecular visualization system, PyMOL^5^ (Schrodinger, 2015).

## Results

### Proteome-wide profiling of *Treponema pallidum* SS14

**Figure 1:**
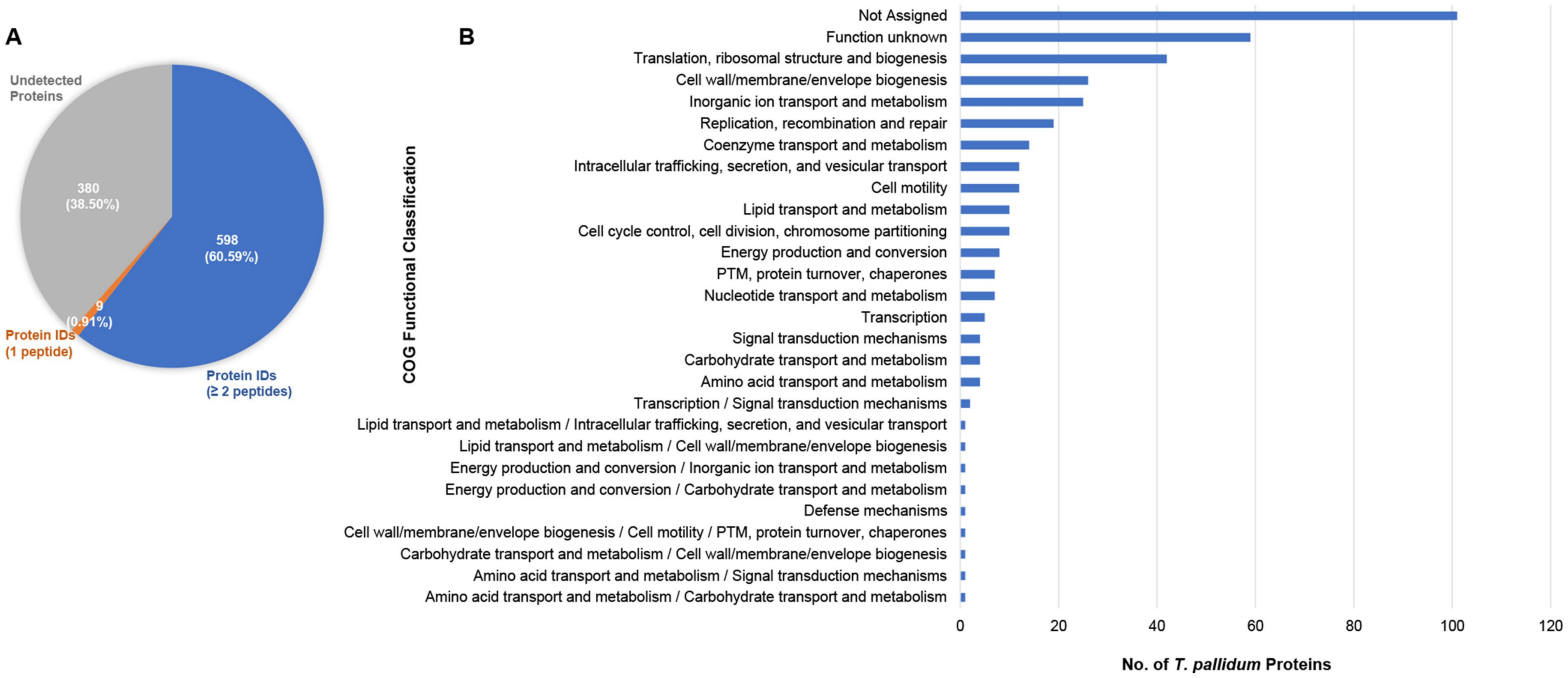
Proteome coverage of *T. pallidum* SS14. **(A)** Pie chart showing the total number of SS14 proteins detected in the present study based on the detection of a single peptide, or at least two peptides. The number of undetected *T. pallidum* proteins is also shown. Values in parentheses indicate the percentage of *T. pallidum* proteins found in each of the three categories. **(B)** Bar chart showing the COG (Clusters of Orthologous Genes) functional classification distribution of 380 *T. pallidum* proteins that were not detected in the present global proteomics study. COG functional classifications were generated by the eggNOG-mapper tool. Not assigned – proteins where eggNOG-mapper was unable to assign functional classifications. PTM: Post-translational modification.

In the present study, 7,163 unique *T. pallidum* peptides were identified which allowed for the detection of 607 of 987 proteins within treponemes from the SS14 strain, representing 61.5% of the total proteome (Figure 1A, Table 1, and Supplementary Table S2). Among these proteins, nine were identified based on the detection of single peptides, whereas 598 were identified based on the detection of at least two peptides (Figure 1A, Table 1, and Supplementary Table S2). Detailed MS data and Scaffold peptide reports for *T. pallidum* SS14, including contaminant peptides and proteins, are provided in Supplementary Table S2.

**Table 1:** Summary of *T. pallidum* SS14 proteins detected.

Number of detected *T. pallidum* SS14 proteins		
Number of proteins detected	1 peptide for protein ID	2 or more peptides for protein ID
607 (61.5% proteome coverage)	9 (0.91% proteome coverage)	598 (60.6% proteome coverage)
Proteome coverage of *T. pallidum* SS14: this study and previous study (Romeis et al., 2021)		
Number of proteins detected	1 peptide for protein ID	2 or more peptides for protein ID
663 (67.2% proteome coverage) (259 SS14 proteins identified only in the present study)	7 (0.71% proteome coverage)	656 (66.6% proteome coverage) (252 SS14 proteins identified only in the present study)
Detection of *T. pallidum* miniproteins of unknown function		
Number of miniproteins detected	1 peptide for protein ID	2 or more peptides for protein ID
14 (17.9% miniprotein coverage) (14 SS14 miniproteins uniquely detected in the present study)	1 (1.3% miniprotein coverage)	13 (16.7% miniprotein coverage) (13 SS14 miniproteins uniquely detected in the present study)
Detection of hypothetical proteins and proteins of unknown function		
Number of proteins detected	1 peptide for protein ID	2 or more peptides for protein ID
146/282 total proteins detected (51.8% coverage) (76 SS14 proteins of unknown function uniquely detected in the present study)	1 (0.4% coverage)	145 (51.4% coverage) (75 SS14 proteins of unknown function uniquely detected in the present study)
Detection of known or predicted OMPs		
Number of proteins detected	1 peptide for protein ID	2 or more peptides for protein ID
22 (64.7% known/predicted OMP coverage) (11 SS14 OMPs uniquely detected in the present study)	0 (0% known/predicted OMP coverage)	22 (64.7% known/predicted OMP coverage) (11 SS14 OMPs uniquely detected in the present study)
Detection of putative pathogenesis-related proteins (PPRPs)		
Number of proteins detected	1 peptide for protein ID	2 or more peptides for protein ID
23 (67.6% coverage) (8 PPRPs identified only in the present study)	0 (0% coverage)	23 (67.6% coverage) (8 PPRPs identified only in the present study)

### Undetected *Treponema pallidum* SS14 proteins

The expression of 380 *T. pallidum* proteins remained undetectable, constituting 38.5% of the treponemal proteome (Figure 1A, Supplementary Tables S2, S3). More than one quarter of these proteins were annotated as proteins of unknown function (Supplementary Tables S2, S3). In line with these results, 42% of undetected proteins were categorized as ‘function unknown’ or were unassignable via COG (Clusters of Orthologous Genes) (Tatusov et al., 1997; Cantalapiedra et al., 2021) analysis (Figure 1B, and Supplementary Table S3). Similar to our previous *T. pallidum* Nichols proteomics studies (Houston et al., 2023; Houston et al., 2024), proteins classified with ‘translation’ and ‘inorganic ion transport and metabolism’ functions were also among the COG classes with the highest number of undetected proteins (Figure 1B, and Supplementary Table S3).

### Data integration: combined coverage of the *Treponema pallidum* SS14 proteome

**Figure 2:**
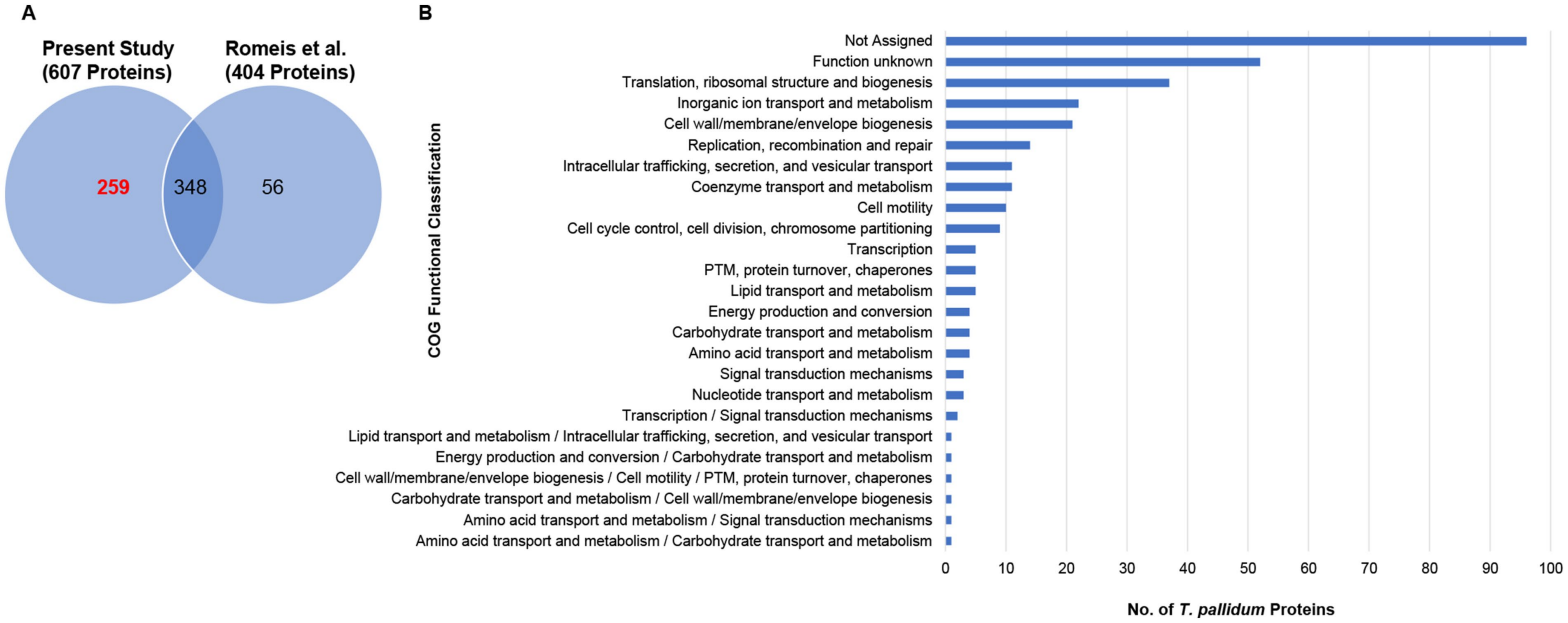
Combined proteome coverage of *T. pallidum* SS14. **(A)** Venn diagram showing the total number of shared and exclusive protein detections corresponding to *T. pallidum* proteins detected in the present study and in a previous SS14 genetic engineering study (Romeis et al., 2021). The total number of *T. pallidum* proteins detected only in the current study (based on the detection of at least one tryptic peptide per identification) is highlighted in red text. **(B)** Bar chart showing the COG functional classification distribution of 324 treponemal proteins not detected in the present study or in the previous SS14 genetic engineering study (Romeis et al., 2021). COG functional classifications were generated by the eggNOG-mapper tool. Not assigned – proteins that were unable to be assigned functional categories by eggNOG-mapper. PTM: Post-translational modification.

In a previous study that performed mass spectrometry analysis to confirm genetic engineering of *T. pallidum*, 404 proteins were detected from *in vitro*-grown *T. pallidum* SS14 (Romeis et al., 2021) that are also annotated in the reference proteome used here (Figure 2A, and Supplementary Table S4). Our analysis from the current study detected an additional 259 proteins from *T. pallidum* SS14 (Figure 2A, Table 1, and Supplementary Table S4). Data integration of our MS findings with the MS-related results from the genetic engineering study performed by Romeis et al. (2021) extended the SS14 proteome coverage to 663 proteins, or 67.2% (Figure 2A, Table 1, and Supplementary Table S4).

### Data integration: undetected proteins from the combined SS14 proteome coverages

After data integration of the MS-related results from the prior study performed by Romeis et al. (2021) with our current results, 324 (32.8%) *T. pallidum* proteins from the SS14 strain remained undetected (Supplementary Table S5). Almost one third of the undetected proteins have no clearly assigned functions in the proteome (Supplementary Table S5). Consistent with this observation, almost half of the 324 undetected proteins could not be classified into functional categories through COG analysis (Tatusov et al., 1997; Cantalapiedra et al., 2021) (Figure 2B and Supplementary Table S5). One third of the undetected proteins were miniproteins, defined as being comprised of 150 amino acids or less (Houston et al., 2022), with an average size of 99 amino acids (Supplementary Table S5). The high number of undetected miniproteins is likely due to their physicochemical properties that limit their detection via MS (Houston et al., 2023; Houston et al., 2024).

### Expression-based comparative analyses of *Treponema pallidum* SS14 and Nichols strains

**Figure 3:**
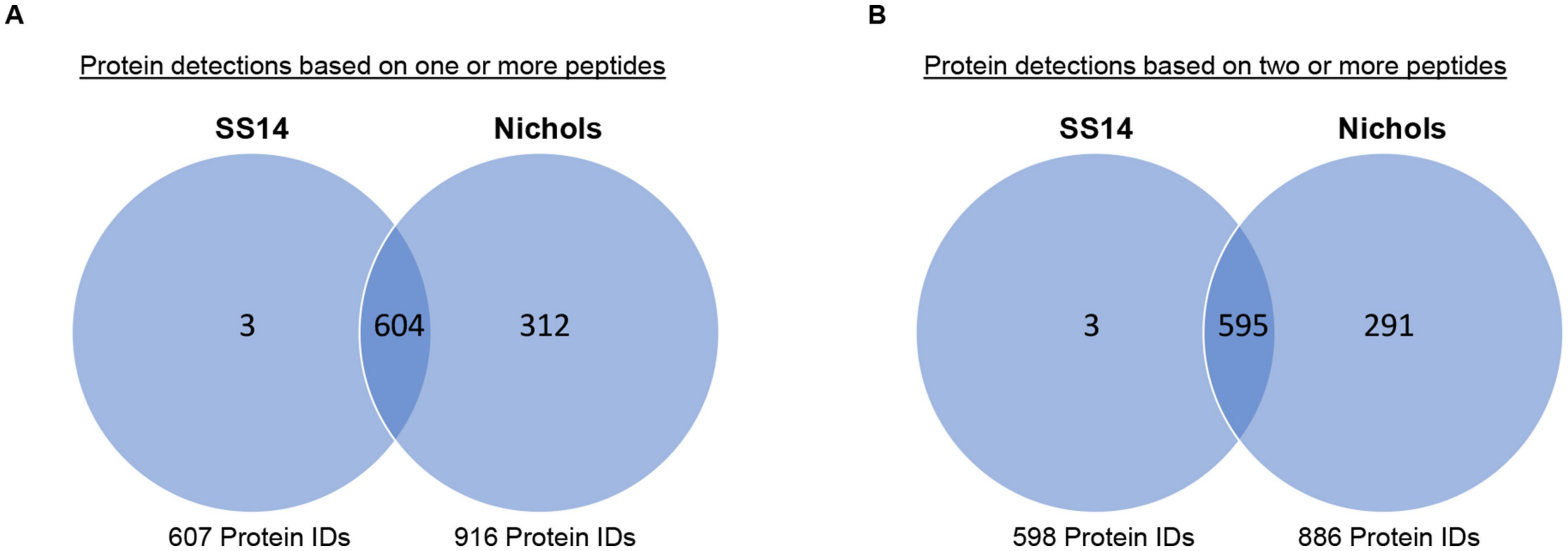
Comparison of *T. pallidum* SS14 and Nichols proteome-wide expression profiles. Venn diagrams showing the number of shared and exclusive protein detections from SS14 and Nichols strains (Houston et al., 2024) based on the detection of **(A)** at least one tryptic peptide or **(B)** two or more tryptic peptides.

Six hundred four treponemal proteins were detected in both the present SS14 study and in our previous Nichols strain study (Houston et al., 2024) based on the detection of at least one peptide (Figure 3A and Supplementary Table S6). When protein identifications were based on the detection of at least two peptides, five hundred ninety-five treponemal proteins were detected in both *T. pallidum* strains (Figure 3B and Supplementary Table S6). Only three proteins were detected exclusively in strain SS14: TPASS_20517 (crossover junction endodeoxyribonuclease, RuvC), TPASS_20849 (50S ribosomal protein L35), and TPASS_ RS02370 (PQQ-like beta-propeller repeat protein) (Figure 3 and Supplementary Table S6). The 291 treponemal proteins that were detected only in the Nichols strain, based on the detection of at least two peptides, are listed in Supplementary Table S6 with annotated functions listed in Supplementary Table S2.

### Quantitation-based comparative analyses of SS14 and Nichols strains

**Figure 4:**
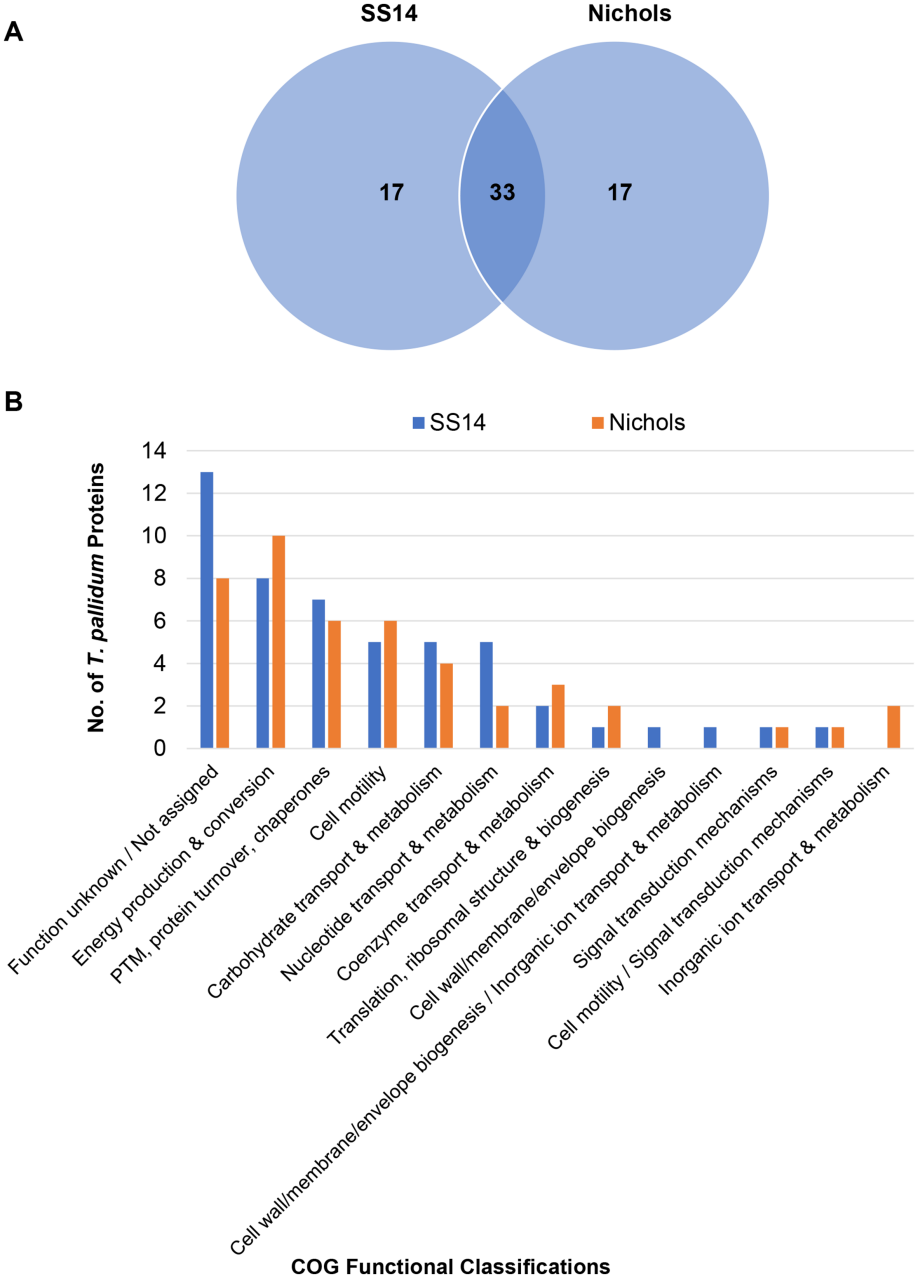
Comparative analyses of the top 50 highest abundant proteins from *T. pallidum* SS14 and Nichols. **(A)** Venn diagram showing the total number of shared and exclusive protein detections corresponding to the top 50 highest abundant proteins from both *T. pallidum* strains. **(B)** Bar chart depicting the COG-based functional classification distribution of the top 50 highest abundant proteins from both *T. pallidum* strains. COG functional classifications were generated by the eggNOG-mapper tool. Not assigned – proteins where eggNOG-mapper was unable to assign functional classifications. PTM: Post-translational modification.

Label-free quantification (LFQ) analysis, based on total spectral counts, allowed for the relative quantification of 598 *T. pallidum* proteins within the SS14 strain and 914 *T. pallidum* proteins within the Nichols strain from our previous study (Houston et al., 2024) (Supplementary Table S7). High abundance proteins (proteins with LFQ intensity values greater than the mean average) from both strains are listed in Supplementary Table S8. Ninety-five percent of all high abundance proteins in the SS14 strain were also detected as high abundance proteins in treponemes from the Nichols strain (Supplementary Table S8), and 33 of the top 50 proteins with the highest expression levels were found in both strains (Supplementary Table S9 and Figure 4A). Most high abundance proteins were assigned functions related to ‘homeostasis’, ‘metabolism’, and ‘protein translation’ in both strains (Supplementary Tables S8, S9; Figure 4B; Supplementary Figure S1).

**Figure 5:**
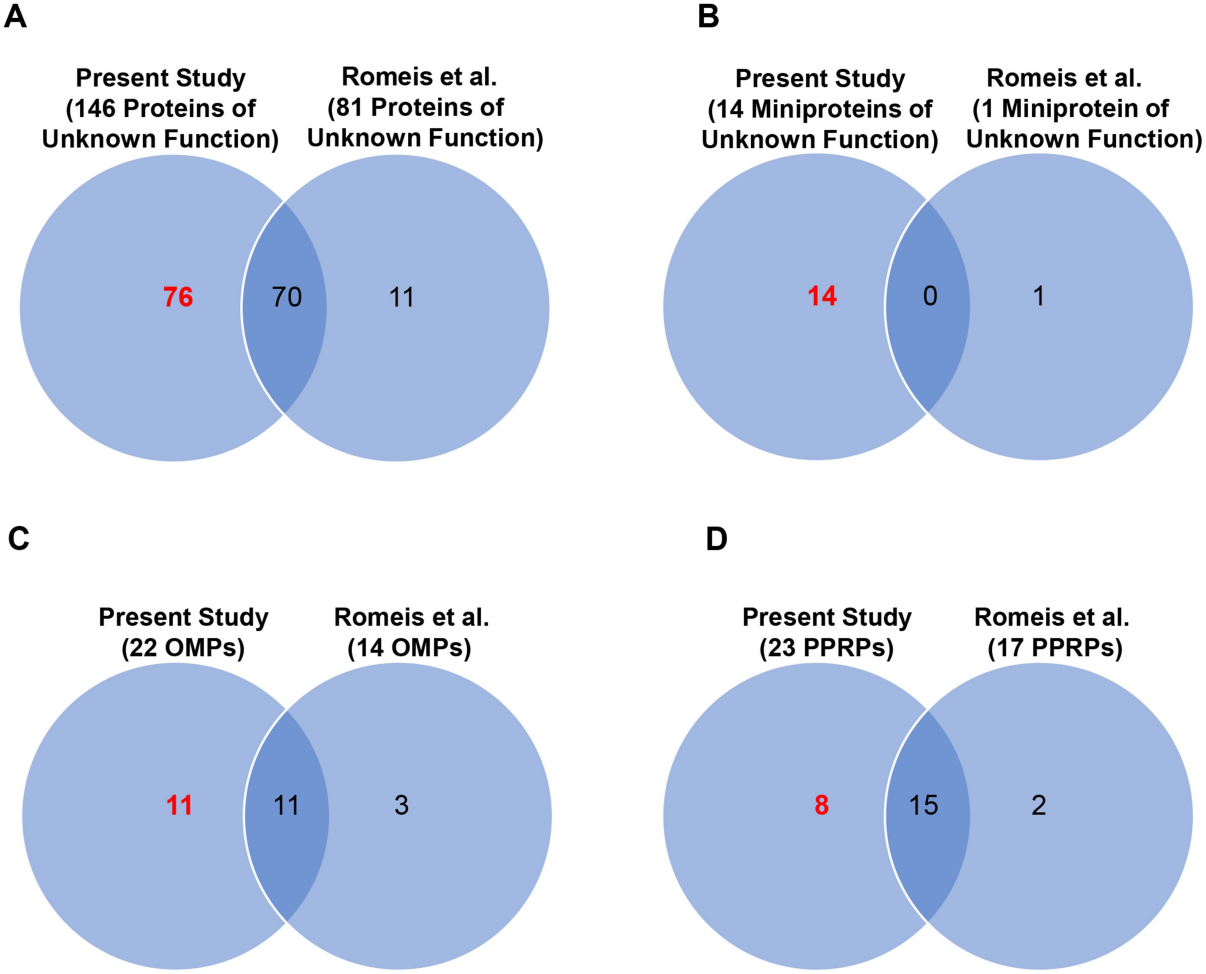
Combined proteome coverage for *T. pallidum* SS14 protein classes of interest. Venn diagrams showing the total number of shared and exclusive protein detections corresponding to **(A)** *T. pallidum* proteins of unknown function (no size restriction), **(B)** *T. pallidum* miniproteins of unknown function, **(C)** known/predicted OMPs, and **(D)** PPRPs, detected in a previous genetic engineering study (Romeis et al., 2021) and in the present MS-based proteomics studies. The total number of *T. pallidum* proteins of interest detected only in the current study (based on the detection of at least one tryptic peptide per identification) is highlighted in red text.

We detected expression of 146 of the 282 proteins from *T. pallidum* SS14 that have no clearly assigned functions in the proteome, including 76 proteins of unknown function whose expression was detected for the first time in this strain (Figure 5A; Table 1; Supplementary Table S10). All 146 proteins of unknown function detected in *T. pallidum* SS14 were also detected in the Nichols strain (Houston et al., 2024) (Supplementary Tables S6, S11). Additionally, proteins with no assigned function that are annotated as either “hypothetical proteins” or DUF (domain of unknown function)-containing proteins were found to comprise less than 10% of the high abundance proteins from both strains (Supplementary Table S8). In the SS14 strain, we also detected expression of 14 of the 78 miniproteins of unknown function (Figure 5B; Table 1; Supplementary Table S12), all of which were also detected in the Nichols strain (Houston et al., 2024) (Supplementary Tables S6, S11). Only one miniprotein of unknown function, TPASS_20777/TPANIC_0777, was detected with high expression levels in both strains (Supplementary Table S8).

Previously, we compiled a list of 34 known and putative OMPs from the Nichols strain from literature reports (Houston et al., 2023). Here we detected 22 of the 34 proteins from SS14, including 11 proteins detected for the first time in this strain (Figure 5C, Tables 1, 2). One putative OMP was detected in both *T. pallidum* strains as a high abundance protein: the “hypothetical protein” TPASS_20855/TPANIC_0855 (Supplementary Table S8). Only one other potential OMP from the Nichols strain was detected as a high abundance protein: the UPF0164 family protein, TPANIC_0858 (Supplementary Table S8). The LFQ rankings for known/putative OMPs ranged from 78 to 579 out of a total of 598 SS14 strain proteins and 71–890 out of a total of 914 Nichols strain proteins (Supplementary Figure S2; Supplementary Table S7).

**Table 2:** MS-based detection of predicted/known OMPs from *T. pallidum* SS14.

OMP locus tag	NCBI functional annotation	MS detection
TPASS_RS05540 (WP_014505382)	Major outer sheath N-terminal domain-containing protein (TprA, N- and C termini truncated)	ND
TPASS_20011	Major outer sheath C-terminal domain-containing protein (TprB)	Present, Romeis
TPASS_20117	Major outer sheath N-terminal domain-containing protein (TprC)	Present
TPASS_20126	Hypothetical protein	ND
TPASS_20131	Major outer sheath N-terminal domain-containing protein (TprD)	Present
TPASS_20155	M23 family metallopeptidase	ND
TPASS_20313	Major outer sheath N-terminal domain-containing protein (TprE)	Present
TPASS_20316	Major outer sheath N-terminal domain-containing protein (TprF)	ND
TPASS_20324	Translocation/assembly module TamB domain-containing protein	Present
TPASS_20326	Outer membrane protein assembly factor BamA	Present, Romeis
TPASS_20421	Tetratricopeptide repeat protein	Present
TPASS_20479	DUF2715 domain-containing protein	Present
TPASS_20483	Fibronectin type III domain-containing protein	ND
TPASS_20515	LPS-assembly protein LptD	Present
TPASS_20548	UPF0164 family protein	Romeis
TPASS_20557	DUF1007 family protein	Romeis
TPASS_20620	Major outer sheath N-terminal domain-containing protein (TprI)	ND
TPASS_20621	Major outer sheath N-terminal domain-containing protein (TprJ)	Present
TPASS_20698	DUF2715 domain-containing protein	ND
TPASS_20733	Outer membrane beta-barrel protein	ND
TPASS_20751	Vascular adhesin/metalloprotease pallilysin	Present, Romeis
TPASS_20855	Hypothetical protein	Present
TPASS_20856	UPF0164 family protein	Romeis
TPASS_20858	UPF0164 family protein	Present, Romeis
TPASS_20859	UPF0164 family protein	Present
TPASS_20865	UPF0164 family protein	Present
TPASS_20897	MSP porin (TprK)	Present, Romeis
TPASS_20923	PEGA domain-containing protein	ND
TPASS_20952	Alpha/beta fold hydrolase	Present, Romeis
TPASS_20966	Hypothetical protein	Present, Romeis
TPASS_20967	Hypothetical protein	Present, Romeis
TPASS_20968	Hypothetical protein	Present, Romeis
TPASS_RS04790	hypothetical protein	Present, Romeis
TPASS_21031	Major outer sheath N-terminal domain-containing protein (TprL)	Present, Romeis

ND, Not detected; Present: Protein detected in the present Study. Romeis: Protein detected in a previous genetic engineering study by Romeis et al. (2021).

We also detected 23 predicted pathogenesis-related proteins (PPRPs) (Cejkova et al., 2012; Petrosova et al., 2012; Petrosova et al., 2013; Houston et al., 2018) from *T. pallidum* SS14, including eight proteins detected for the first time in this strain (Figure 5D; Tables 1, 3). Most PPRPs from both strains were found to be of low relative abundance, with only five PPRPs from SS14 and seven PPRPs from Nichols detected as high abundance proteins (Supplementary Table S8). In addition, four of the high abundance proteins were shared between SS14 and Nichols (Supplementary Table S8). The single, highly expressed PPRP from SS14 that was not detected as highly abundant in the Nichols strain was TPASS_20594 (DUF2147-containing protein) (Supplementary Table S8). This protein was previously identified as a structural ortholog of a *Helicobacter pylori* protein that functions in host colonization and persistence (Houston et al., 2018).

**Table 3:** MS-based detection of PPRPs from *T. pallidum* SS14.

PPRP locus tag	NCBI functional annotation	MS detection
TPASS_20020	vWA domain-containing protein	Present, Romeis
TPASS_20027	Hemolysin family protein	ND
TPASS_20028	Hemolysin family protein	ND
TPASS_20126	Hypothetical protein	ND
TPASS_20134	Hypothetical protein	Present, Romeis
TPASS_20225	Leucine-rich repeat domain-containing protein	Present, Romeis
TPASS_20246	VWA domain-containing protein	ND
TPASS_20262	Crp/Fnr family transcriptional regulator	Present
TPASS_20399	Flagellar basal-body MS-ring/collar protein FliF	Present, Romeis
TPASS_20401	Flagellar assembly protein FliH	Present, Romeis
TPASS_20402	Flagellar protein export ATPase FliI	Present, Romeis
TPASS_20421	Tetratricopeptide repeat protein	Present
TPASS_20544	SpnA family nuclease	Present, Romeis
TPASS_20579	Hypothetical protein	ND
TPASS_20594	DUF2147 domain-containing protein	Present
TPASS_20598	Hypothetical protein	Present
TPASS_20625	Hypothetical protein	Present
TPASS_20649	Hemolysin family protein	Present, Romeis
TPASS_20714	Flagellar biosynthesis protein FlhA	Present
TPASS_20715	Flagellar biosynthesis protein FlhB	Present
TPASS_20733	Outer membrane beta-barrel protein	ND
TPASS_20783	Hypothetical protein	ND
TPASS_20789	Outer membrane lipoprotein-sorting protein	Present, Romeis
TPASS_20854	SpoIIE family protein phosphatase	Present
TPASS_20862	FKBP-type peptidyl-prolyl cis-trans isomerase	Present, Romeis
TPASS_20911	FlhB-like flagellar biosynthesis protein	ND
TPASS_20928	Hypothetical protein	Present, Romeis
TPASS_20936	Hemolysin family protein	Romeis
TPASS_20966	Hypothetical protein	Present, Romeis
TPASS_20967	Hypothetical protein	Present, Romeis
TPASS_20968	Hypothetical protein	Present, Romeis
TPASS_RS04790	Hypothetical protein	Present, Romeis
TPASS_21033	Patatin-like phospholipase family protein	Romeis
TPASS_21037	Hemolysin III family protein	ND

ND, Not detected; Present: Protein detected in the present Study. Romeis: Protein detected in a previous genetic engineering study by Romeis et al. (2021).

### Identification of proteome annotation errors in *Treponema pallidum* SS14

In previous proteomics studies, we identified 23 protein annotation errors from *T. pallidum* Nichols; seven proteins that were incorrectly deleted from the proteome (“deletion errors”), nine proteins that were annotated with prematurely truncated or incorrect N-termini (“N-terminal errors”), and seven proteins for which we detected expression but whose ORFs had been annotated as “pseudo” genes/non-coding ORFs (“pseudo errors”) (Houston et al., 2023; Houston et al., 2024). Here, we identified nine “N-terminal errors,” three “pseudo errors,” and no “deletion errors” in proteins from *T. pallidum* SS14 (Table 4, Supplementary Table S13, and Supplementary Figure S3). Similar to the Nichols strain (Houston et al., 2023; Houston et al., 2024), most of the errors found in the SS14 proteome were associated with proteins of unknown function (Table 4 and Supplementary Table S13). Only two of the 12 SS14 proteome annotation errors were previously identified in the Nichols proteome (Houston et al., 2023; Houston et al., 2024) (Table 4 and Supplementary Table S13). This low overlap in inter-strain proteome annotation errors is partially due to the fact that 12 of the 23 proteins from the Nichols strain with annotation errors (Houston et al., 2024) were either not detected in the present SS14 study, or the proteins were not present in the SS14 proteome annotation. These findings indicate a SS14 proteome annotation error rate of at least 1.2% which is comparable to the Nichols proteome annotation error rate of 2.3% (Houston et al., 2024), when adjusted for proteome coverage.

**Table 4:** Proteome annotation errors identified in *T. pallidum* SS14.

Locus tag	Functional annotation	NCBI proteome annotation errors (NCBI reference sequence NC_021508, March 2023 annotation)
TPASS_20185	Signal peptidase I	Incorrectly truncated N-terminus
TPASS_20491	Endolytic transglycosylase MltG	Incorrectly truncated N-terminus
TPASS_20496	Tetratricopeptide repeat protein	Incorrectly truncated N-terminus
TPASS_20535	Hypothetical protein	Incorrectly truncated N-terminus
TPASS_20648	Tetratricopeptide repeat protein	Incorrectly truncated N-terminus
TPASS_20675	TraB/GumN family protein	Incorrectly truncated N-terminus
TPASS_20776	ComF family protein	Incorrectly truncated N-terminus
TPASS_20938	Hypothetical protein	Incorrectly truncated N-terminus
TPASS_20978	Signal peptidase II	Incorrectly truncated N-terminus
TPASS_RS05380 (equivalent to TPANIC_0868)	Flagellin	“Pseudo” (non-coding annotation)
TPASS_20897	MSP porin (TprK)	“Pseudo” (non-coding annotation)
TPASS_RS04790 (equivalent to TPANIC_0969)	Hypothetical protein	“Pseudo” (non-coding annotation)

### Data integration analyses: identification of SS14-Nichols inter-strain protein sequence differences

**Figure 6:**
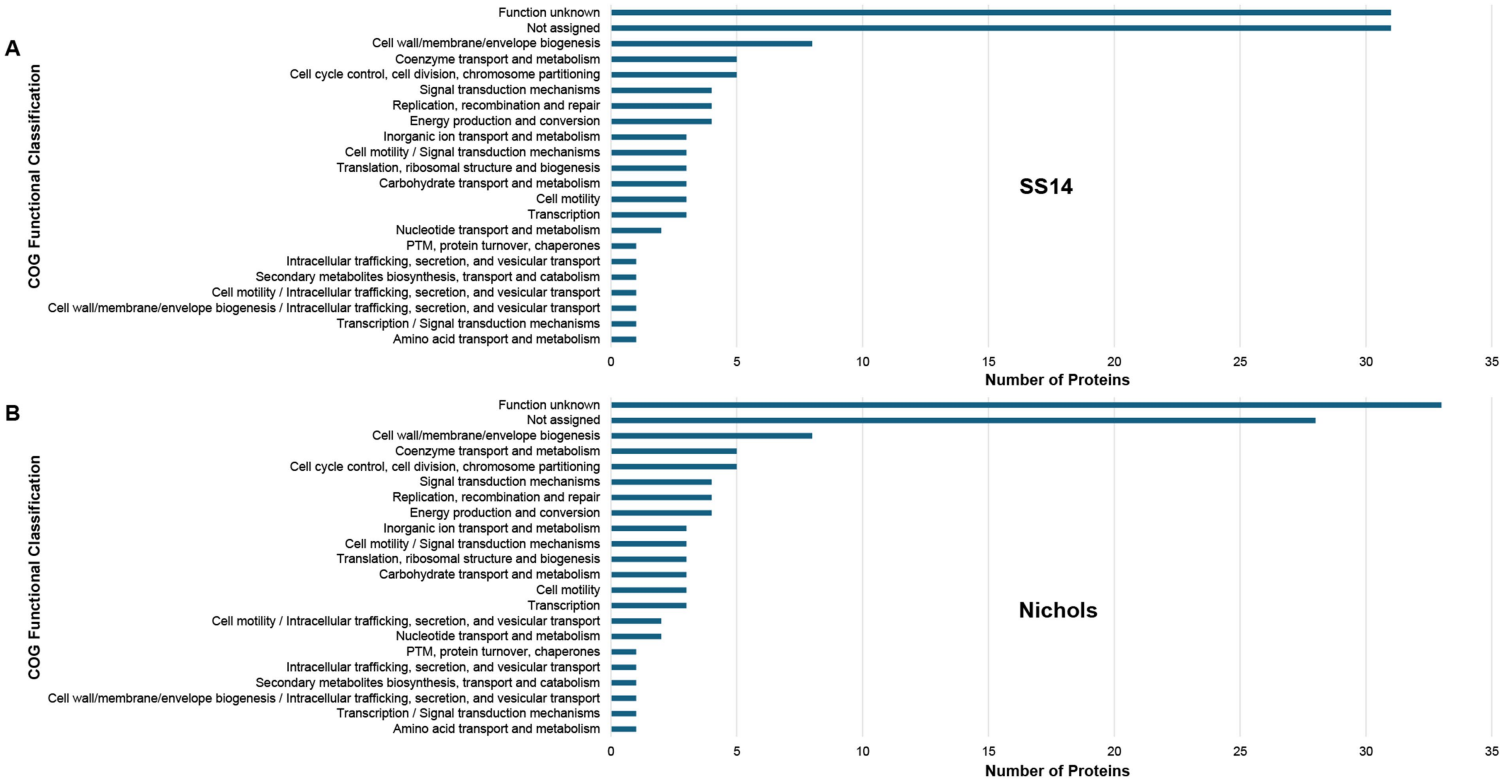
Functional classification of 119 variable proteins from *T. pallidum* SS14 and Nichols strains. Bar charts showing the COG functional classification distribution of **(A)** 119 treponemal proteins from *T. pallidum* SS14, and **(B)** the corresponding 119 *T. pallidum* Nichols proteins, that are annotated with at least one amino acid difference between the two strains. COG functional classifications were generated by the eggNOG-mapper tool. Not assigned – proteins that were unable to be assigned functional categories by eggNOG-mapper. PTM: Post-translational modification. Experimental data was derived from the present and previous studies (Osbak et al., 2016; Romeis et al., 2021; Houston et al., 2023; Houston et al., 2024).

Using proteome-wide annotation comparative analyses, we identified 119 proteins with at least one amino acid difference between the corresponding proteins from SS14 and Nichols strains (Supplementary Figure S4 and Supplementary Table S14). Half of these 119 proteins were identified as proteins of unknown function (Figure 6 and Supplementary Table S14), 10 were previously identified as PPRPs (Cejkova et al., 2012; Petrosova et al., 2012; Petrosova et al., 2013; Houston et al., 2018) (Table 3 and Supplementary Table S14), and seven were identified as miniproteins of unknown function. Two of the miniproteins were previously identified as potential antimicrobial peptides (AMPs) in the Nichols strain (Houston et al., 2022) (Supplementary Tables S12, S14). Furthermore, 19 known/putative OMPs were found to be annotated with at least one amino acid difference between the two strains (Table 5).

**Table 5:** SS14 and Nichols OMP inter-strain sequence differences and experimental validations.

*Tp* SS14 Locus Tag	Annotated Function	Annotated Inter-Strain Variable Amino Acids (IVAA)	IVAA Detected^a^
TPASS_RS05540	Major outer sheath N-terminal domain-containing protein, TprA	42 amino acid N-terminal truncation	No
TPASS_20117	Major outer sheath N-terminal domain-containing protein, TprC	10 amino acid substitutions	2/10
TPASS_20131	Major outer sheath N-terminal domain-containing protein, TprD	117 amino acid substitutions 4 amino acid insertions 6 amino acid gaps	0/127
TPASS_20313	Major outer sheath N-terminal domain-containing protein, TprE	1 amino acid substitution	No
TPASS_20316	Major outer sheath N-terminal domain-containing protein, TprF	5 amino acid N-terminal extension	No
TPASS_20326	Outer membrane protein assembly factor BamA	9 amino acid substitutions	5/9
TPASS_20479	DUF2715 domain-containing protein	17 amino acid N-terminal truncation	No
TPASS_20515	LPS-assembly protein LptD	10 amino acid substitutions	0/10
TPASS_20548	UPF0164 family protein	25 amino acid substitutions 1 amino acid insertion	0/26
TPASS_20620	Major outer sheath N-terminal domain-containing protein, TprI	8 amino acid substitutions	No
TPASS_20621	Major outer sheath N-terminal domain-containing protein TprJ	2 amino acid substitutions	0/2
TPASS_20858	UPF0164 family protein	1 amino acid substitution	1/1
TPASS_20865	UPF0164 family protein	2 amino acid substitutions 1 amino acid insertion	1/3 (1 amino acid substitution)
TPASS_20897	MSP porin, TprK	101 amino acid substitutions	0/101
TPASS_20952	Alpha/beta fold hydrolase	1 amino acid substitution	0/1
TPASS_20966	Hypothetical protein	3 amino acid substitutions	0/3
TPASS_20968	Hypothetical protein	1 amino acid substitution	1/1
TPASS_RS04790	Hypothetical protein	8 amino acid N-terminal extension^b^ 6 amino acid substitutions 9 amino acid gaps	No
TPASS_21031	Major outer sheath N-terminal domain-containing protein, TprL	25 amino acid substitutions	4/25

a Number of IVAAs that were confirmed in SS14 and Nichols strains via the detection of corresponding peptides in both strains (present study and Osbak et al., 2016; Romeis et al., 2021; Houston et al., 2023; Houston et al., 2024).

b SS14 protein annotated as a Pseudo ORF (N-terminal extension does not contain a start site residue).

IVAA, Annotated Inter-Strain Variable Amino Acids.

No, peptides with IVAAs were not detected.

IVAA descriptions relate to the SS14 proteins in comparison to the corresponding Nichols strain proteins.

### Data integration analyses: experimental validation of SS14-Nichols inter-strain protein sequence differences

Data integration analyses using proteome-wide protein expression comparative analyses, which incorporated experimental data from the present study and previous studies (Osbak et al., 2016; Romeis Lieberman et al., 2021; Houston et al., 2023; Houston et al., 2024), confirmed the expression of inter-strain protein sequence differences in 32 of the 119 proteins (Supplementary Figure S4 and Supplementary Table S14). These analyses also confirmed amino acid sequence differences for six of the 19 known/putative *T. pallidum* OMPs that are annotated with at least one amino acid difference between the two strains (Table 5). Protein tertiary structure modeling using AlphaFold 3 (see footnote 3) (Abramson et al., 2024) demonstrated that most of these inter-strain OMP differences, including the six experimentally validated amino acid differences, were predicted to occur within surface-exposed regions of the OMPs that are located at the interface between the bacterium and the host (LaFond and Lukehart, 2006) (Supplementary Figure S5).

## Discussion

The Nichols and SS14 strains of *T. pallidum* are both highly virulent. However, SS14 strains exhibit a higher frequency of macrolide resistance (Stamm et al., 1988; Stamm and Bergen, 2000), and based on currently available clinical sampling data are the more prevalent infecting strain worldwide (Arora et al., 2016; Beale et al., 2021; Lieberman et al., 2021; Sena et al., 2024). Although previous proteomics studies have detected 95% of all proteins in Nichols/Nichols-like strains (McGill et al., 2010; Osbak et al., 2016; Houston et al., 2023; Houston et al., 2024), in-depth proteome-wide analyses of SS14 strains had not been performed prior to this study.

In the present study, we characterized the proteome of *T. pallidum* SS14 via the detection of 61.5% of proteins from this strain. Quantitative comparative analyses revealed similar protein abundance profiles between the SS14 and Nichols strains, for both the total proteome coverage and across specific protein functional classes of interest. These included OMPs and potential pathogenesis related proteins, which in agreement with previous *T. pallidum* studies (Izard et al., 2009; Liu et al., 2010; Radolf and Kumar, 2017; Houston et al., 2023; Houston et al., 2024) generally exhibited low-level expression. This low abundance of known and putative OMPs may partially contribute to the ability of both *T. pallidum* strains to evade the immune response and establish and maintain persistent infection (Cameron, 2005). Given that the SS14 and Nichols mass spectrometry analyses were temporally independent, statistical analyses of the LFQ results could not be performed and analyses were limited to relative quantification comparisons based solely on LFQ rankings.

Bacterial OMPs, located at the interface between the bacterium and the host, are targets of protective antibodies during infection (LaFond and Lukehart, 2006) and therefore have been identified as leading syphilis vaccine candidates (Giacani et al., 2010; Lithgow et al., 2017; Haynes et al., 2021; Delgado et al., 2022). The analyses of *T. pallidum* proteomes can aid in syphilis vaccine development by confirming inter-strain protein expression of vaccine candidates, including OMPs, since universal expression among strains is a necessary feature of a vaccine candidate. The present study detected expression of 11 OMPs, including several vaccine candidates, for the first time in *T. pallidum* SS14. Proteomic analyses can also confirm annotated protein sequence variations among strains, including within treponemal OMPs. A previous *T. pallidum* genome sequencing study reported that inter-strain sequence variability occurs mostly within the predicted surface-exposed loops of four known/putative OMPs being investigated as vaccine candidates (TP_0326, TP_0548, TP_0966, and TP_0967) (Lieberman et al., 2021). In the present study, comparative proteomic analyses showed that an additional 16 known/putative OMPs have inter-strain amino acid sequence differences, with the differences being confirmed in six known/putative *T. pallidum* OMPs via the analyses of MS data from the present and past studies (Osbak et al., 2016; Romeis et al., 2021; Houston et al., 2023; Houston et al., 2024). Furthermore, protein tertiary structure modeling showed that these inter-strain amino acid differences were found to be located primarily within predicted surface-exposed regions in the 16 additional OMPs. Several of these treponemal OMPs with surface-exposed inter-strain variable amino acids play key roles in *T. pallidum* pathogenesis, and have been identified as current syphilis vaccine candidates, including TPASS_20326/TPANIC_0326 (BamA) (Cameron et al., 2000; Luthra et al., 2015), TPASS_20858/TPANIC_0858 (UPF0164 family protein) (Hawley et al., 2021; Delgado et al., 2022), TPASS_20897/TPANIC_0897 (TprK) (Centurion-Lara et al., 2000; Giacani et al., 2010; Parveen et al., 2019), and TPASS_21031/TPANIC_1031 (TprL) (Haynes Lieberman et al., 2021). In addition, potential B cell epitopes (BCEs) have been identified in the literature that include some of the surface-exposed inter-strain variable amino acids reported here: these include BCE3 and BCE8 of TPANIC_0515 (Hawley et al., 2021), and BCE1 and BCE3 of TPANIC_0548 (Hawley et al., 2021). The detection and localization of these inter-strain variable residues within OMPs can aid subunit-based vaccine design by allowing identification of surface-exposed regions of the proteins that are conserved across circulating *T. pallidum* strains.

The present study has limitations. A relatively high number of undetected proteins were observed in the SS14 strain compared to the Nichols strain (Houston et al., 2024), which limited the comparative analyses that could be performed. Possible explanations for the higher number of undetected proteins in SS14 include: (1) the presence of more post-translational modifications (PTMs) within proteins found in the SS14 strain which could hinder MS detection due to the generation of tryptic peptides with observed masses that differ from the expected masses; or (2) lower protein expression levels within treponemes from the SS14 strain. Another limitation, which is inherent to MS analyses, is that lack of protein detection does not equate to lack of protein expression; instead, proteins may not be detected due to incompatibility with MS methodology. Therefore, it is possible that the undetected proteins in this study may indeed be expressed by *T. pallidum*. However, it should be noted that identical sample preparation methods were used to prepare samples for proteomic analyses for both the Nichols and SS14 strains, suggesting the lower protein detection coverage within treponemes from the SS14 strain occurred due to biological rather than technical reasons. The use of the optimized proteomics protocol from the previously performed Nichols proteomic study may represent a further limitation of the study, since this resulted in a single sample being analyzed by MS for SS-14 compared to the three samples analyzed for Nichols (Houston et al., 2024). A final limitation is that treponemes used in the present study were cultured under *in vitro* conditions, and thus protein expression may not be fully representative of circulating strains.

In conclusion, the present study reveals the protein repertoire expressed by the SS14 *T. pallidum* strain during *in vitro* growth, and provides the first comparative analyses of protein expression and quantitation profiles of the reference strains from each of the two *T. pallidum* phylogenetic lineages. This knowledge can be used to inform syphilis vaccine design by confirming the expression of leading vaccine candidates in a clinical *T. pallidum* strain (SS14), and via the mapping and confirmation of annotated inter-strain amino acid sequence variances in leading vaccine candidates from SS14 and Nichols strains. The data presented in the study are deposited in the MassIVE repository, accession number MSV000095818 (ProteomeXchange identifier PXD055753).

## Data Availability

The datasets presented in this study can be found in online repositories. The names of the repository/repositories and accession number(s) can be found in the article/Supplementary material.

## Footnotes

^1^ https://www.thegpm.org/crap/

^2^ https://www.ncbi.nlm.nih.gov/research/cog

^3^ https://deepmind.google/technologies/alphafold/

^4^ https://services.healthtech.dtu.dk/services/SignalP-6.0/

^5^ https://pymol.org/
