# Supplementary figures and images for: Proteomic analysis of the Treponema pallidum subsp. pallidum SS14 strain: coverage and comparison with the Nichols strain proteome
Source: Front Microbiol. 2024 Dec 11;15:1505893. doi: 10.3389/fmicb.2024.1505893 (PMC11668736; doi:10.3389/fmicb.2024.1505893)

# Supplementary Figure S1

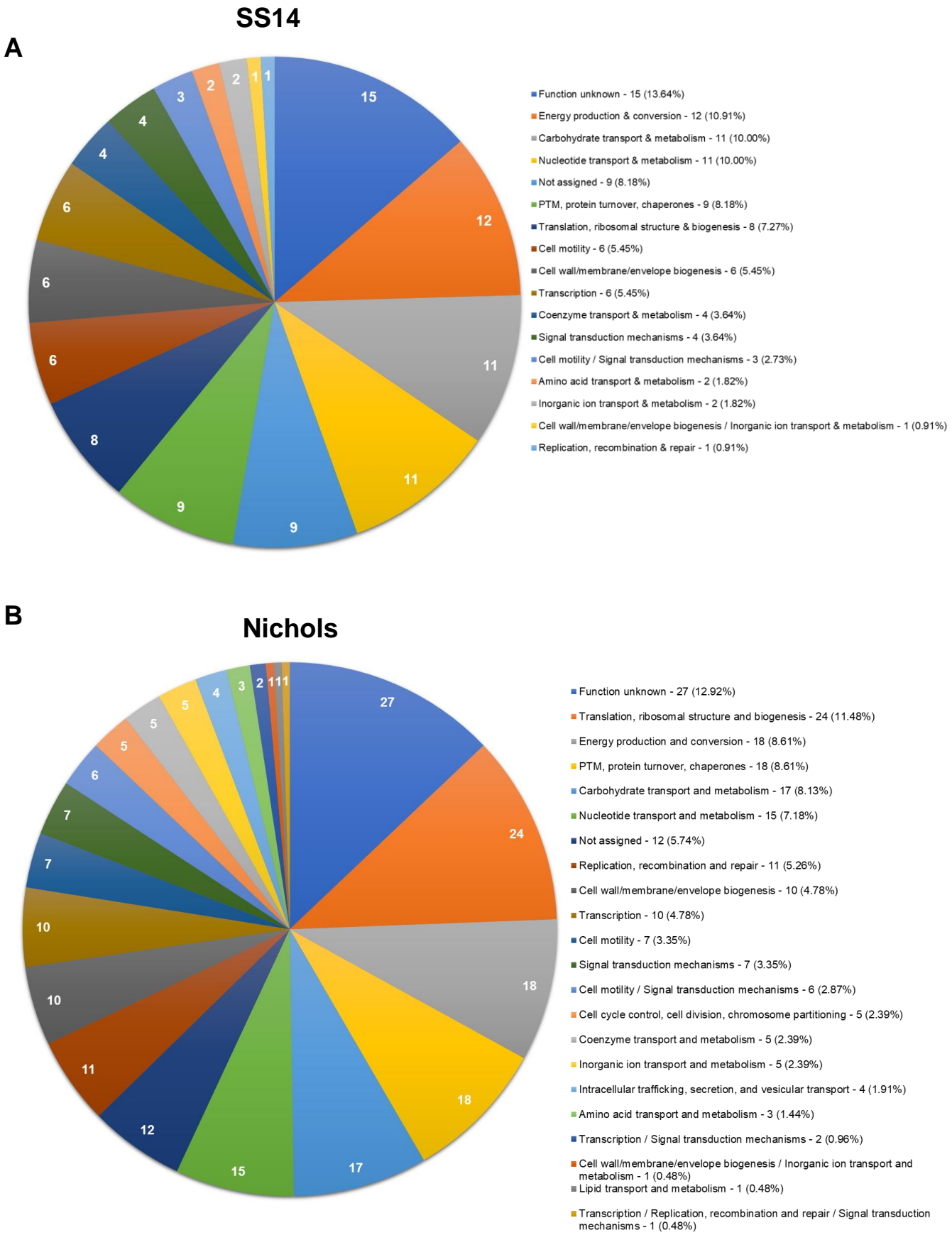

Supplement: Supplementary file 1 [file Data_Sheet_1.PDF]
