# Supplementary material for: Proteomic analysis of the Treponema pallidum subsp. pallidum SS14 strain: coverage and comparison with the Nichols strain proteome
Source: Front Microbiol. 2024 Dec 11;15:1505893. doi: 10.3389/fmicb.2024.1505893 (PMC11668736; doi:10.3389/fmicb.2024.1505893)
Supplement: Supplementary file 2 [file Data_Sheet_2.PDF]

Supplementary Figure S2

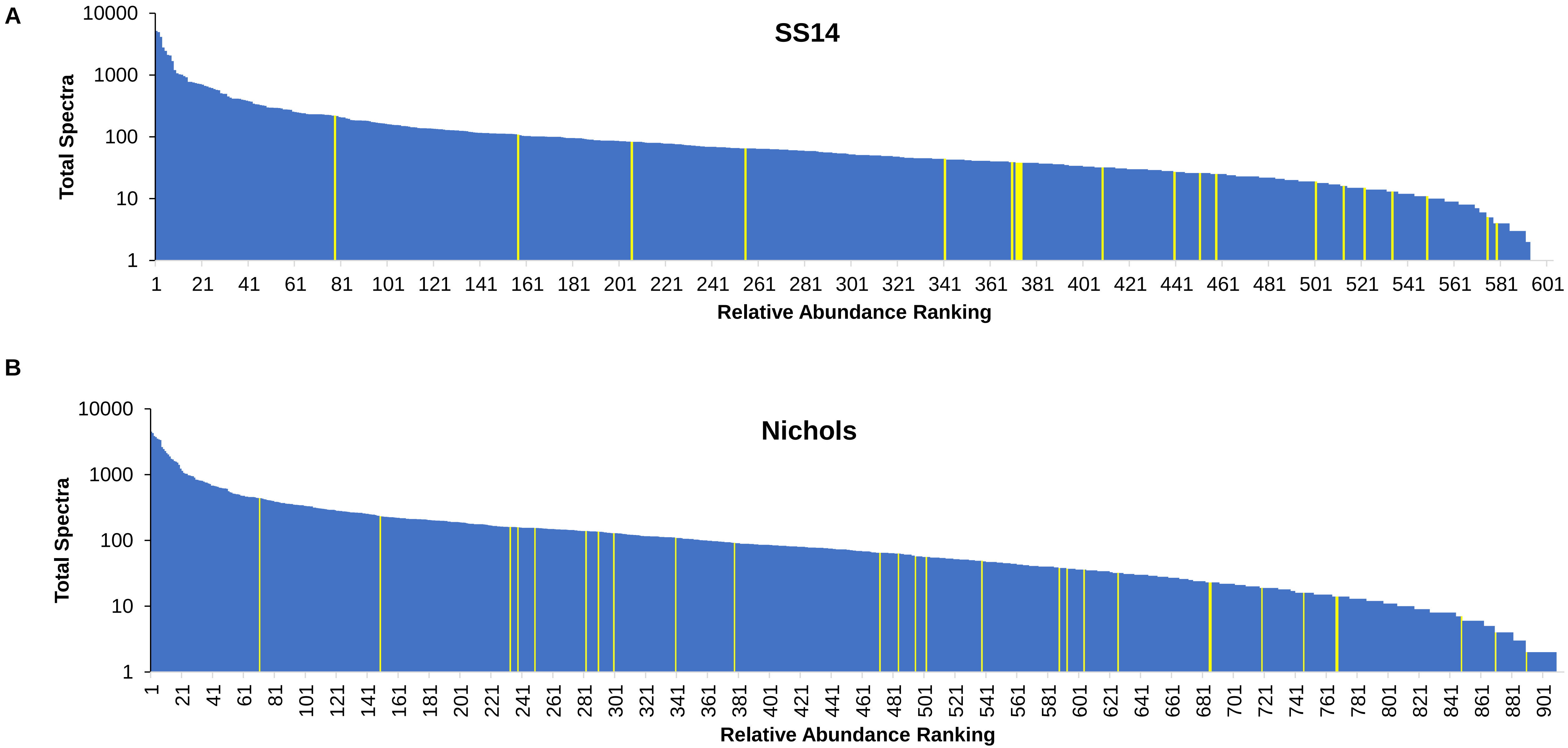

Supplementary Figure S2. Relative abundances of *T. pallidum* OMPs from SS14 and Nichols strains. (A) Graph showing the relative abundances of 598 SS14 proteins (ranked from highest to lowest relative abundance [x-axis]), including 20 known/predicted OMPs (yellow highlighting). Only protein detections that were derived from the *T. pallidum* SS14 database search in the present study were used for LFQ analyses. (B) Same as (A) with 914 Nichols proteins (including 28 known/predicted OMPs). Higher total spectra values correspond to higher protein abundance.
