# Supplementary material for: Proteomic analysis of the Treponema pallidum subsp. pallidum SS14 strain: coverage and comparison with the Nichols strain proteome
Source: Front Microbiol. 2024 Dec 11;15:1505893. doi: 10.3389/fmicb.2024.1505893 (PMC11668736; doi:10.3389/fmicb.2024.1505893)
Supplement: Supplementary file 3 [file Data_Sheet_3.PDF]

Supplementary Figure S3

A

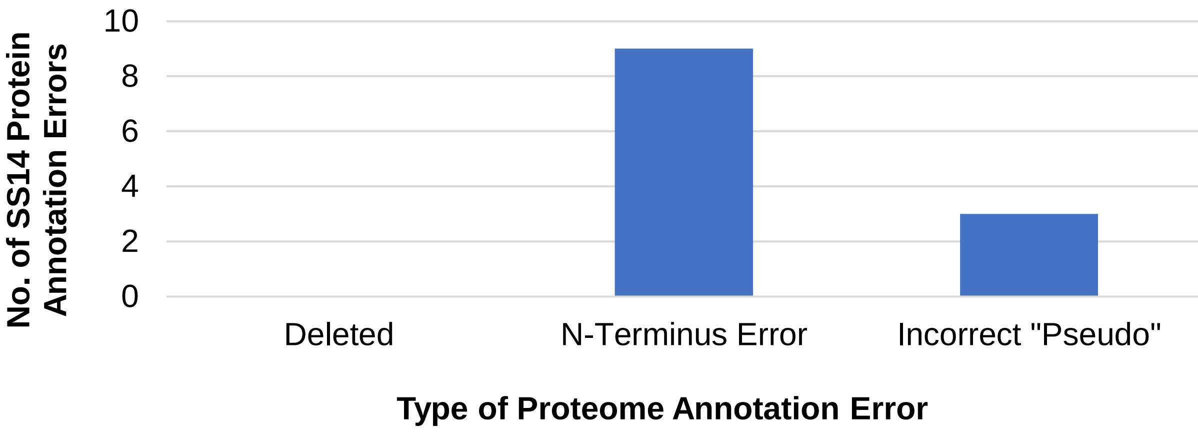

B

TPASS_20185

MFARWRRYSYLARREARRNATAVCSAGVGFFLFYLFITTHVVAAYRIQADSMQPTLSAGDCVLASSLFRFARIK
RGDLVLATPLEKEDIGLFKRAMNAVLGFASLQLYRPFGAADRMFSRPQMRRVVGLPGDTVYMRDFVLYVKPHGQ

TPASS_20491

MSIRTFSFTPSVRLRRIVLWGSLFCAGVLCLLCLCLLVGLAPVRPFVKKEHMFTVQSGVGARKVIHELRNARLI
RSEWAARLYVFARALNFKAGSYAVSPAMSAVRILTMLDDVEQQRFIKVTVPEGLTVKKIAALLQDATVVSAAAF

TPASS_20496

MEFLLRRSGHQSFFPGRRKLPNSVFLFSVFFALVVGVGVGAWRYRRYYRGLPSARSVYEDWKNGNYKAVYDKAA
EILQRRVFDAEMLALHGFAAYYIFSEQTDLSVSYDYLNSAIVSLRRALHVVRPAEVPNVSYVLGKAYYQRGYYY

TPASS_20535

MSAAWVGNMDKGVMVRLAEVEDAAAVLVEKAQEQAQRMRESAEAAGETLVKEAEGSVVEAQEQAWHEACARIDA
QRREACGQLERELSVLEEDVERFEAFLDGFFFG

TPASS_20648

MYLSCGVIPSLHVYLSGHRGKGKSMKGLLCVYYGALCSIVFAQTGVQLYEAGRKAHVQEDWHAAIEFYQEALKK
NASYNLAYRGLAECFYALGEYDQALHHVRKAQKLMAQDLSLEKLCAFSLVGQGELDQARSLFEEILARYPNDVD

TPASS_20675

MNTTGRPVFPLLRRTVLKRCSLCATRCAIVFLCVLLILPFLSCCTSLSRGALPSLISHKERMFWEIRGPQGSVY
ILGTISVGSEKLLHFQDKILDVFDSASRLYAELGSEDIKNFASVLQRRMLHGMLEQENAAPTLSSLSREELEML

TPASS_20776

MILFSRARLWARDGVARAYVSFLGPRRCVFCASRCTGTVPLCCSCVQDRLYARAHDFLEHPEDFCSRCAKPLVS
ARALCVSCRALRESGETPALWRVFSLLPYLGVGRPLMSLWKTQQERNFDALFSRIAGCFLRTARERSFVTASTE

TPASS_20938

MKFCRGWRGARAGQFARLLCVCWCSLGACSCVRESTERSGVVPISPSSQQKVLFVFGKKYSGHVYLVDCVERVC
EAWRMRARVVEYGDLTRLSALPRMKVVLEAVHEMRPDAVVSLGLPVGGGKYLLAVKRERAQTLIVSLLPEEEVL

TPASS_20978

MIRSRPLRVDTMKLTRIQKEKWIPLFAAGLVVVLDQCAKLLVGAYVPTNTSGVRVLGDFVRIVHVYNVGAAFSI
GHQLNQVLRTLVLGIVPLIIMFLIVFSYFRTDAFCPVQRWAVSGIIGGGIGNLIDRFLRPNGVLDFIDVKFFGI

Supplementary Figure S3. *Treponema pallidum* SS14 proteome annotation errors. (A) Bar graph summarizing the number of *T. pallidum* proteins that contain one of the three different annotation error types. (B) Amino acid sequences of nine *T. pallidum* SS14 proteins that were annotated with prematurely truncated N-termini. Peptides from previous (pre-2023) NCBI annotated versions of each of the nine proteins were detected in the present study (underlined/yellow background). Blue arrows/residues with green backgrounds indicate the start sites of the incorrectly truncated protein versions in the NCBI March 2023 proteome annotation. C-terminal amino acid sequences have not been included here due to space constraints.
