# Supplementary material for: Proteomic analysis of the Treponema pallidum subsp. pallidum SS14 strain: coverage and comparison with the Nichols strain proteome
Source: Front Microbiol. 2024 Dec 11;15:1505893. doi: 10.3389/fmicb.2024.1505893 (PMC11668736; doi:10.3389/fmicb.2024.1505893)
Supplement: Supplementary file 4 [file Data_Sheet_4.PDF]

Supplementary Figure S4

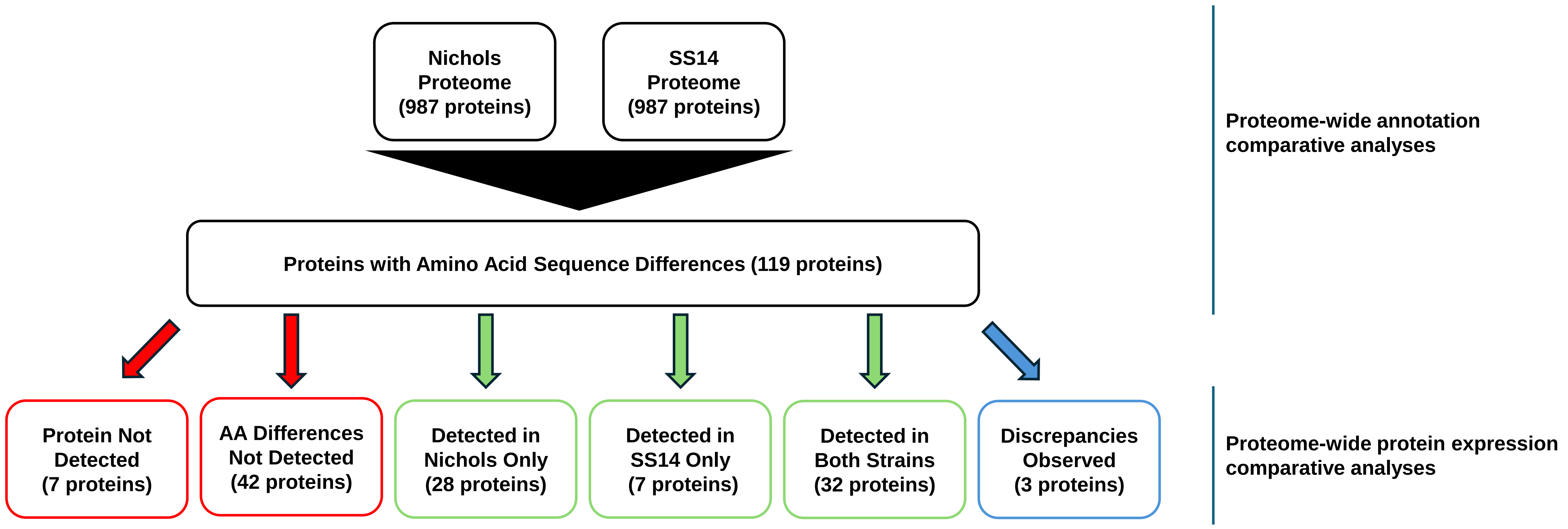

Supplementary Figure S4. Data integration and comparative analyses of proteomes from *T. pallidum* strains Nichols and SS14. Proteome-wide annotation comparative analyses were performed to identify all treponemal proteins from the two NCBI proteomes that are annotated with at least one amino acid difference between the two strains. Proteome-wide protein expression comparative analyses were then used to validate as many inter-strain amino acid annotation differences as possible using experimental data from the present and previous studies (Osbak et al., 2016; Romeis et al., 2021; Houston et al., 2023; Houston et al., 2024).
