# Supplementary material for: Proteomic analysis of the Treponema pallidum subsp. pallidum SS14 strain: coverage and comparison with the Nichols strain proteome
Source: Front Microbiol. 2024 Dec 11;15:1505893. doi: 10.3389/fmicb.2024.1505893 (PMC11668736; doi:10.3389/fmicb.2024.1505893)
Supplement: Supplementary file 5 [file Data_Sheet_5.PDF]

Supplementary Figure S5

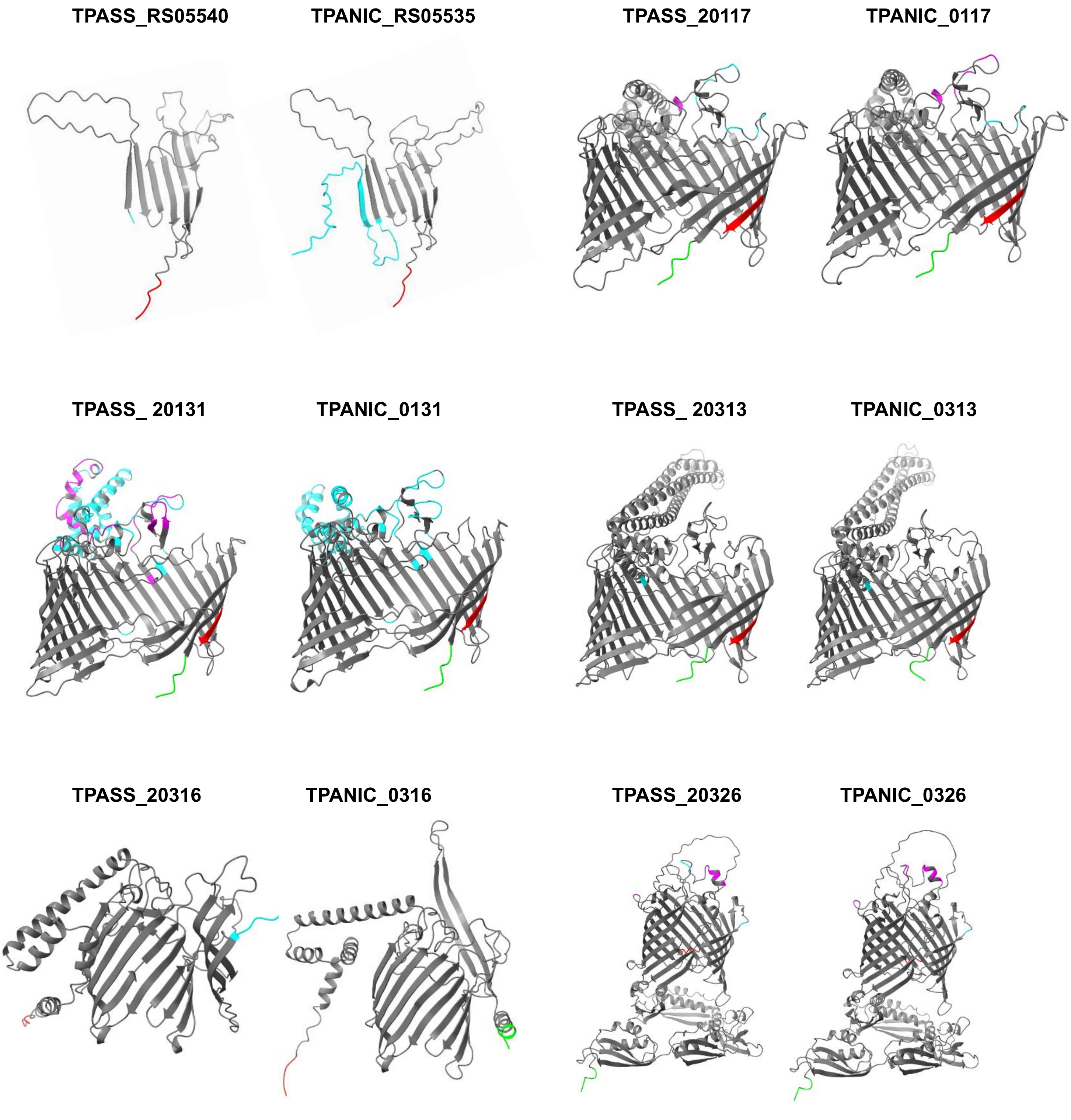

TPASS_20479

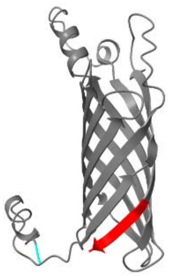

TPANIC_0479

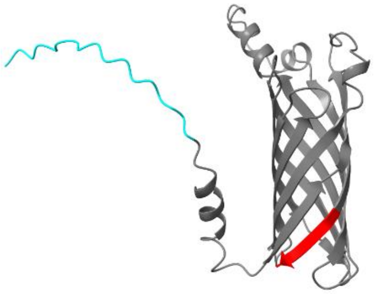

TPASS_20515

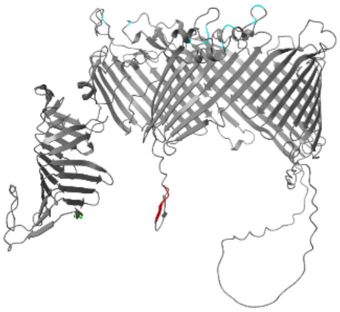

TPANIC_0515

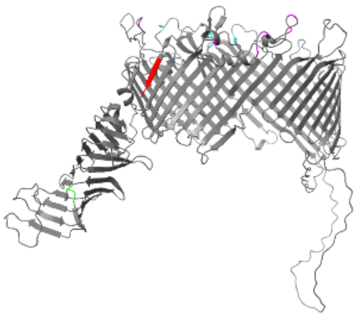

TPASS_20548

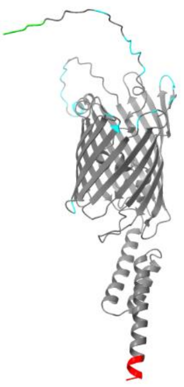

TPANIC_0548

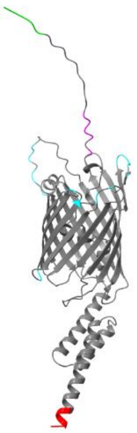

TPASS_ 20620

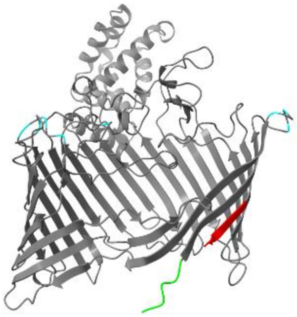

TPANIC_0620

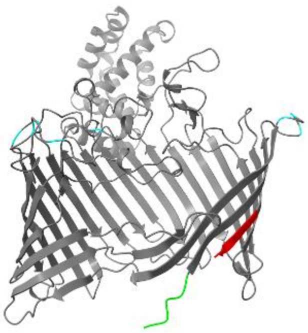

TPASS_20621

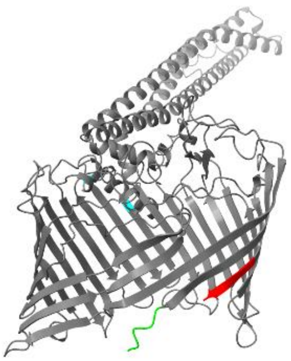

TPANIC_0621

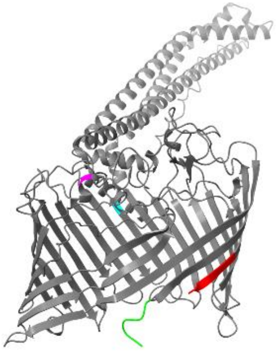

TPASS_20858

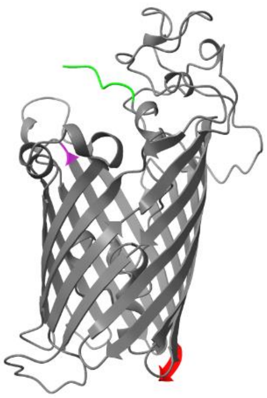

TPANIC_0858

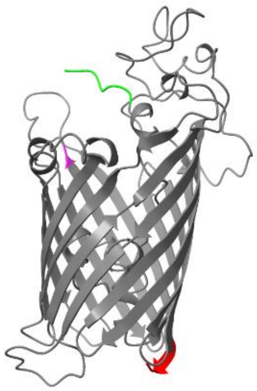

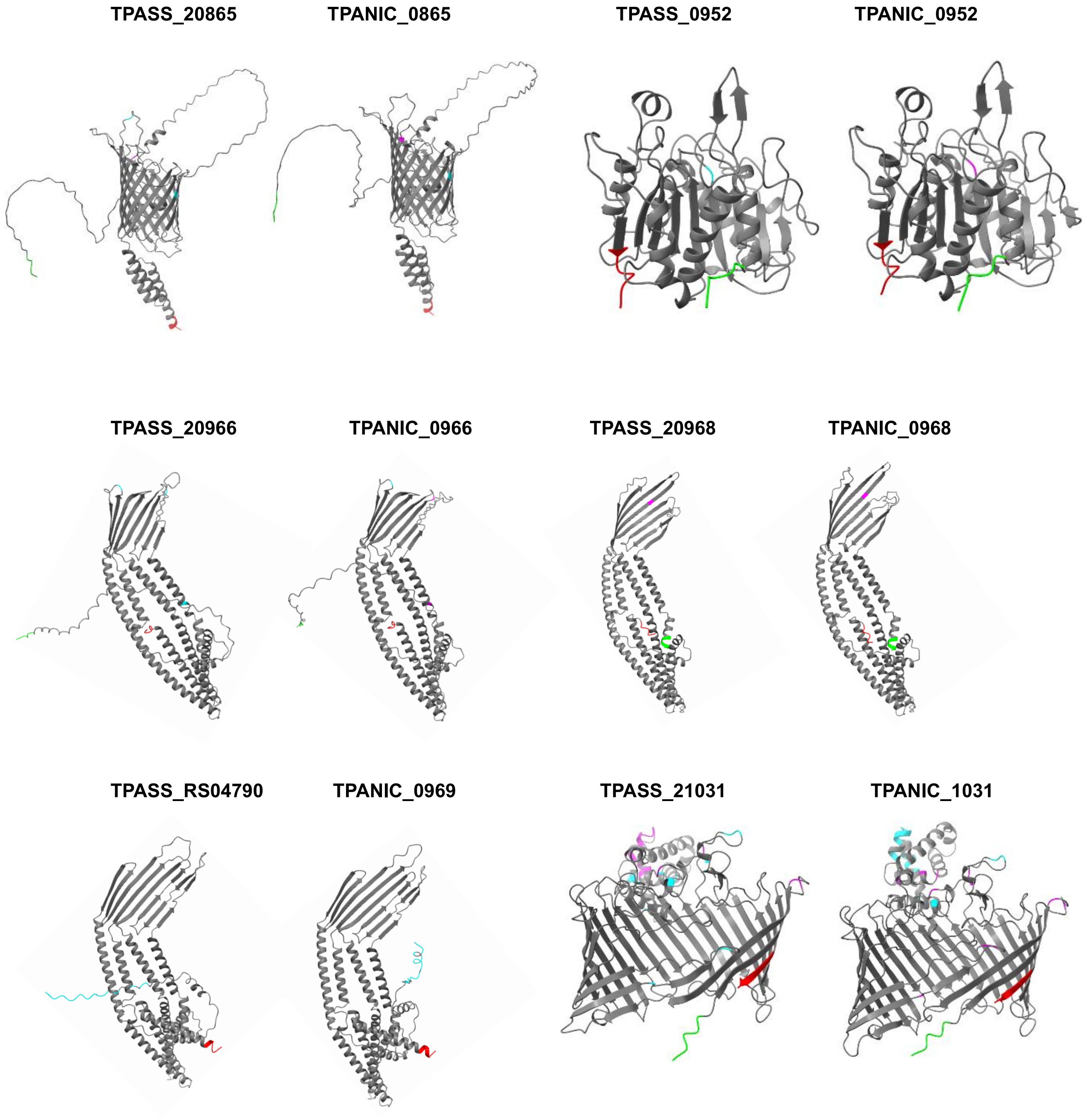
TPASS_20865
TPANIC_0865
TPASS_0952
TPANIC_0952
TPASS_20966
TPANIC_0966
TPASS_20968
TPANIC_0968
TPASS_RS04790
TPANIC_0969
TPASS_21031
TPANIC_1031

TPASS_20897 (X→G) **TPANIC_0897 (X→G)**

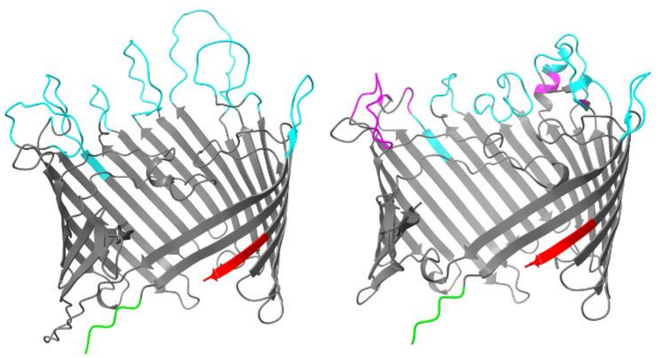

Supplementary Figure S5. Structure models of 19 *T. pallidum* putative/known OMPs with SS14/Nichols inter-strain amino acid differences. Highest-confidence AlphaFold 3 structure models of 19 putative/known OMPs with at least one inter-strain amino acid sequence difference annotated in the proteomes of *T. pallidum* SS14 and Nichols. SS14 proteins are shown on the left of each protein pair (Locus tag: TPASS). Nichols proteins are shown on the right of each protein pair (Locus tag: TPANIC). Protein structure models are orientated with predicted surface-exposed loop regions at the top of each model. Green: first five N-terminal amino acids. Red: last five C-terminal amino acids. Magenta: inter-strain variable amino acids detected in present or past studies (Osbak et al., 2016; Romeis et al., 2021; Houston et al., 2023; Houston et al., 2024). Cyan: inter-strain variable amino acids not detected in present or past studies (Osbak et al., 2016; Romeis et al., 2021; Houston et al., 2023; Houston et al., 2024). SignalP 6.0 was used to identify N-terminal signal peptides, which were removed from applicable protein sequences prior to structure modeling. The predicted signal peptide (SpI) for TPASS_20316 was not removed as the variable amino acids were present in this region. For the TPASS_20897/TPANIC_0897 (TprK) protein pair, within-strain variable amino acids residues (X) were replaced with glycine (G).
