# Supplementary material for: Proteomic analysis of the Treponema pallidum subsp. pallidum SS14 strain: coverage and comparison with the Nichols strain proteome
Source: Front Microbiol. 2024 Dec 11;15:1505893. doi: 10.3389/fmicb.2024.1505893 (PMC11668736; doi:10.3389/fmicb.2024.1505893)
Supplement: Supplementary file 6 [file Data_Sheet_6.ZIP › Supplementary Tables/Supplementary_Table_S13.docx]

**Supplementary Table S13. Detailed information for *T. pallidum* SS14 proteome annotation errors.**

| Locus Tag | Functional Annotation | NCBI Proteome Annotation Errors  (NCBI reference sequence NC_021508, March 2023 annotation) |
| --- | --- | --- |
| TPASS_20185 | signal peptidase I | Incorrectly truncated N-terminus  YSYLAR peptide detected in SS14: corresponds to a peptide located in the extended N-terminus of the 2021 version of TPASS_20185 (NCBI locus, WP_010881632)  Extended N-terminus of WP_010881632: MFARWRRYSYLARREARRNATAVCSAG |
| TPASS_20491 | endolytic transglycosylase MltG | Incorrectly truncated N-terminus  TFSFTPSVR peptide detected in SS14: corresponds to a peptide located in the extended N-terminus of the 2021 version of TPASS_20491 (NCBI locus, WP_014342451)  Extended N-terminus of WP_014342451:  MSIRTFSFTPSVRLRRIVLWGSLFCAGVLCLLCLCLLV  GLAPVRPFVKKEH |
| TPASS_20496 | tetratricopeptide repeat protein | Incorrectly truncated N-terminus  SGHQSFFPGR peptide detected in SS14: corresponds to a peptide located in the extended N-terminus of the 2021 version of TPASS_20496 (NCBI locus, WP_063353235)  Extended N-terminus of WP_063353235: MEFLLRRSGHQSFFPGRRKLPNS |
| TPASS_20535 | hypothetical protein | Incorrectly truncated N-terminus  LAEVEDAAAVLVEK peptide detected in SS14: corresponds to a peptide located in the extended N-terminus of the 2017 version of TPASS_20535 (NCBI locus, WP_014342464)  Extended N-terminus of WP_014342464: MSAAWVGNMDKGVMVRLAEVEDAAAVLVEKAQEQAQR |
| TPASS_20648 | tetratricopeptide repeat protein | Incorrectly truncated N-terminus  MYLSCGVIPSLHVYLSGHR peptide detected in SS14: corresponds to a peptide located in the extended N-terminus of the 2021 version of TPASS_20648 (NCBI locus, WP_010882093)  Extended N-terminus of WP_010882093: MYLSCGVIPSLHVYLSGHRGKGKS |
| TPASS_20675 | TraB/GumN family protein | Incorrectly truncated N-terminus  GALPSLISHKER peptide detected in SS14: corresponds to a peptide located in the extended N-terminus of the 2021 version of TPASS_20675 (NCBI locus, WP_010882120)  Extended N-terminus of WP_010882120: MNTTGRPVFPLLRRTVLKRCSLCATRCAIVFLCVLLILPFL  SCCTSLSRGALPSLISHKER |
| TPASS_20776 | ComF family protein | Incorrectly truncated N-terminus  CVFCASR and CTGTVPLCCSCVQDR peptides detected in SS14: corresponds to two peptides located in the extended N-terminus of the 2021 version of TPASS_20776 (NCBI locus, WP_010882221)  Extended N-terminus of WP_010882221: MILFSRARLWARDGVARAYVSFLGPRRCVFCASRCTGTVPLCCSC |
| TPASS_20938 | hypothetical protein | Incorrectly truncated N-terminus  SGVVPISPSSQQK peptide detected in SS14: corresponds to a peptide located in the extended N-terminus of the 2021 version of TPASS_20938 (NCBI locus, WP_010882381)  Extended N-terminus of WP_010882381: MKFCRGWRGARAGQFARLLCVCWCSLGACSCVRESTERSG |
| TPASS_20978 | signal peptidase II | Incorrectly truncated N-terminus  MIRSRPLRVDTMK peptide detected in SS14: corresponds to a peptide located in the extended N-terminus of the 2021 version of TPASS_20978 (NCBI locus, WP_012460612)  Extended N-terminus of WP_012460612: MIRSRPLRVDT  N-terminus of TPASS_20978 (2024 version) has been revised to: MIRSRPLRVDT |
| TPASS_RS05380  (equivalent to TPANIC_0868) | flagellin | “Pseudo” (non-coding annotation) |
| TPASS_20897 | MSP porin (TprK) | “Pseudo” (non-coding annotation) |
| TPASS_RS04790  (equivalent to TPANIC_0969) | hypothetical protein | “Pseudo” (non-coding annotation) |
